# Investigation of prediction accuracy and the impact of sample size, ancestry, and tissue in transcriptome‐wide association studies

**DOI:** 10.1002/gepi.22290

**Published:** 2020-03-19

**Authors:** James J. Fryett, Andrew P. Morris, Heather J. Cordell

**Affiliations:** ^1^ Population Health Sciences Institute, Faculty of Medical Sciences Newcastle University Newcastle upon Tyne UK; ^2^ Division of Musculoskeletal and Dermatological Sciences University of Manchester Manchester UK

**Keywords:** complex traits, gene expression, transcriptome‐wide association study

## Abstract

In transcriptome‐wide association studies (TWAS), gene expression values are predicted using genotype data and tested for association with a phenotype. The power of this approach to detect associations relies, at least in part, on the accuracy of the prediction. Here we compare the prediction accuracy of six different methods—LASSO, Ridge regression, Elastic net, Best Linear Unbiased Predictor, Bayesian Sparse Linear Mixed Model, and Random Forests—by performing cross‐validation using data from the Geuvadis Project. We also examine prediction accuracy (a) at different sample sizes, (b) when ancestry of the prediction model training and testing populations is different, and (c) when the tissue used to train the model is different from the tissue to be predicted. We find that, for most genes, the expression cannot be accurately predicted, but in general sparse statistical models tend to outperform polygenic models at prediction. Average prediction accuracy is reduced when the model training set size is reduced or when predicting across ancestries and is marginally reduced when predicting across tissues. We conclude that using sparse statistical models and the development of large reference panels across multiple ethnicities and tissues will lead to better prediction of gene expression, and thus may improve TWAS power.

## 1 INTRODUCTION

Transcriptome‐wide association studies (TWAS) are an increasingly popular approach to integrate genome‐wide association study (GWAS) data with pre‐existing gene expression data to understand biological pathways and identify potentially causal genes underlying disease. This methodology uses reference panels with measured gene expression and single‐nucleotide polymorphism (SNP) genotypes to build a predictive model representing the genetically regulated component of gene expression. This model is then applied to GWAS genotype data to impute gene expression values, which can be tested for association with the phenotype of interest. The methodology is implemented in multiple software packages (Barbeira et al., 2018; Bhutani, Sarkar, Park, Kellis, & Schork, 2017; Gamazon et al., 2015; Gusev et al., 2016; Vervier & Michaelson, 2016), and has been used to investigate the role of gene expression in many traits and diseases (Mancuso et al., 2017; Torres et al., 2017).

The power to detect associations in TWAS partially relies on the accuracy of gene expression prediction. To achieve accurate predictions of gene expression, a statistical model whose underlying assumptions best match the true genetic architecture of gene expression should be used. For most genes, the expression is thought to have a sparse architecture, regulated by a few SNPs each with a large effect (Wheeler et al., 2016). A previous comparison of a limited number of statistical methods for gene expression prediction found that sparse models, such as those obtained from least absolute angle and selection operator (LASSO), outperformed more polygenic models, such as those obtained from linear mixed modeling, providing further evidence of a sparse underlying genetic architecture (Zeng, Zhou, & Huang, 2017). Current software packages for TWAS use a range of methods to construct models for expression prediction, including sparse and polygenic methods. The PrediXcan, S‐PrediXcan, and FUSION packages use elastic net (Zou & Hastie, 2005), a sparse form of penalised regression, to model the relationship between genotype and gene expression. Elastic net and other forms of penalised regression, such as LASSO and ridge regression, have been shown to perform well at prediction of a range of complex phenotypes (Bae, Choi, Kim, & Park, 2016; Spiliopoulou et al., 2015; Warren, Casas, Hingorani, Dudbridge, & Whittaker, 2014) and at a prediction of disease risk (Choi, Bae, & Park, 2016; Guo, Wei, Keating, & Hakonarson, 2016; Wei et al., 2013). In addition, FUSION also uses the Bayesian sparse linear mixed model (BSLMM; Zhou, Carbonetto, & Stephens, 2013), and the best linear unbiased predictor (BLUP; Yang et al., 2010) to construct some of its prediction models. The BSLMM is a spike and slab model, which has been shown to perform well at disease risk and complex phenotype prediction (Berger, Perez‐Rodriguez, Veturi, Simianer, & de los Campos, 2015; Moser et al., 2015), while the BLUP, is a polygenic model that has been used widely for the prediction of complex traits in humans and animals (Bermingham et al., 2015; de Los Campos, Hickey, Pong‐Wong, Daetwyler, & Calus, 2013; Yang et al., 2010). Another popular approach for complex trait prediction is the machine learning method Random Forests (Breiman, 2001). While it has not specifically been used for expression prediction, it has shown promising results when used for prediction of other complex phenotypes (Sarkar, Rao, Meher, Nepolean, & Mohapatra, 2015; Xu et al., 2011), making it a good candidate for further exploration. To date, there has been no systematic comparison of all these approaches for gene expression prediction.

A number of other factors are also likely to play a role in determining how accurately gene expression can be predicted from SNP genotypes, including (but not limited to) ancestry, sample size, and tissue matching. Ancestry is known to be important in the prediction of complex traits. Polygenic risk scores estimated in a population of one ancestry typically perform poorly when applied to populations of alternative ancestries (Vilhjalmsson et al., 2015), while some work has suggested that gene expression prediction models trained using a sample of one ancestry tend to perform more poorly when applied to a sample of a different ancestry (Mogil et al., 2018). Another factor known to affect prediction of complex traits is the sample size, with larger sample sizes typically associated with improved prediction accuracy (Dudbridge, 2013, 2016; Wei et al., 2013; Wray et al., 2013). Finally, an issue more specific to gene expression is prediction across tissues. Gene expression prediction models for a specific tissue of interest may not be available (due to a lack of publicly available matched genotype and expression data), and so prediction models for an alternative (proxy) tissue may be used instead. The relevance of results from this proxy tissue to the tissue of interest depends on how well prediction models for the proxy tissue can predict expression in the tissue of interest. Early work suggested that prediction accuracy was indeed reduced when predicting across tissues in this manner (Gamazon et al., 2015). While there has been some previous investigation of some of these factors, more work is needed to truly understand how they affect prediction accuracy in TWAS.

Here, we test the performance of statistical methods that have either been implemented in transcriptome imputation software or that have shown promise in predicting complex phenotypes (LASSO, ridge regression, elastic net, BSLMM, BLUP, and Random Forest) at predicting gene expression levels, using data from the Geuvadis project in which genome‐wide genotype and lymphoblastoid cell line (LCL) gene expression data were measured in 462 individuals of European or African origin. We also compare the performance of these statistical modeling approaches with pre‐existing prediction models from the PrediXcan software package. Finally, we examine the impact of sample size, ancestry and tissue matching on the accuracy of gene expression prediction.

## 2 METHODS

### 2.1 Data and quality control

#### 2.1.1 Geuvadis

The genotype data for 465 individuals (of Northern and Western European [CEU], Finnish [FIN], British [GBR], Tuscan [TSI], or Yoruba [YRI] ancestry) and PEER‐factor normalized gene expression counts of 23,722 genes from LCLs from 462 individuals measured by RNA‐seq were downloaded from the Geuvadis website (Lappalainen et al., 2013). A total of 421 of the samples were genotyped as part of the 1000 Genomes Project, and the remaining samples were genotyped on an Omni 2.5M array and then imputed to the 1000 Genomes Phase 1 reference panel. Samples of CEU, FIN, GBR, and TSI ancestry were combined into a single group of European (EUR) samples. Three EUR individuals for whom genotype data were present, but expression data were not, were removed, leaving 462 samples to be taken forward (comprising 373 EUR samples and 89 YRI samples). EUR and YRI samples were separated before genotype quality control (QC). Within each group, we removed SNPs with minor allele frequency (MAF) < 0.01 or with imputation quality *R* ^2^ < 0.8 in the individuals for whom genotypes were imputed. We also removed SNPs with missing data in any samples. Gene expression data were used as is, with no further processing or quality control after download from the Geuvadis website.

#### 2.1.2 Wellcome Trust Case Control Consortium (WTCCC)

Case/control data for type 1 diabetes (T1D) from WTCCC1 (Wellcome Trust Case Control Consortium, 2007) were obtained. In total, these data comprised 1,963 T1D cases and 2,938 controls. QC was performed, removing SNPs with MAF < 0.01, SNPs with abnormal cluster plots from genotyping and SNPs that failed the original WTCCC1 automated QC procedures. These data were then imputed to the 1000 Genomes Phase 1 reference panel using the Michigan Imputation Server (Das et al., 2016), using ShapeIT prephasing, and the EUR population to check the consistency of allele frequencies. Further QC was performed on these imputed data, again removing SNPs with MAF < 0.01 and any SNPs with imputation *R* ^2^ < 0.8.

### 2.2 Modeling methods

In all methods applied here, gene expression was used as the phenotype (*y*), and genotypes at all SNPs within 1 megabase of the gene start or end site were used as predictors (*x*), with no additional covariates included in the regression.

#### 2.2.1 Ridge regression

Ridge regression (Hoerl & Kennard, 2000) applies an L2 penalty to determine regression coefficients by minimising

$${\left(y-X\beta \right)}^{2}+\lambda \sum_{j=1}^{p}\beta_{j}^{2},$$

where *y* is the gene expression, *X* is a matrix of SNP genotypes, *β* represents regression coefficients, and *λ* is a regularisation parameter. This penalty enables the shrinkage of coefficients to near zero, but not absolute zero, resulting in a polygenic model where many predictors each have small effects on the trait of interest. Here we implemented it using the R package *glmnet*, with *λ* determined via 10‐fold cross‐validation.

#### 2.2.2 Lasso

An alternative penalized regression approach is the LASSO, which instead imposes a L1 type penalty on regression coefficients (Tibshirani, 1996). This penalty seeks to minimise

$${\left(y-X\beta \right)}^{2}+\lambda \sum_{j=1}^{p}|\beta_{j}|.$$

By using an L1 penalty function, the LASSO can shrink some coefficients to absolute zero, resulting in a sparser solution than ridge regression is capable of. Here we implemented LASSO via the R package *glmnet*, with *λ* determined by 10‐fold cross‐validation.

#### 2.2.3 Elastic net

The elastic net (Zou & Hastie, 2005), combines the L1 penalty used by LASSO and the L2 penalty used by ridge regression, giving the following minimization problem:

$${\left(y-X\beta \right)}^{2}+\lambda \left(\frac{1}{2}\left(1-\alpha \right)\sum_{j=1}^{p}\beta_{j}^{2}+\alpha \sum_{j=1}^{p}\left|\beta_{j}\right|\right).$$

By including both penalties, and using the parameter *α* to determine the weight of each penalty, the elastic net enables the degree of model sparsity to vary between that of ridge regression (typically polygenic) and LASSO (typically sparse). In this study, the elastic net was used twice—once with *α* set to .5, and once in which *α* was determined by cross‐validation. The *λ* parameter was determined by cross‐validation in both instances. Here we implemented an elastic net using the R package *glmnet*.

#### 2.2.4 Bayesian sparse linear mixed model

The BSLMM is a spike‐and‐slab prior model that combines a standard linear mixed model (LMM) with a Bayesian variable selection regression approach (BVSR). The LMM aspect of the model assumes that all covariates have a small effect on the trait of interest (polygenic), while the BVSR portion also allows some variants to have a larger effect on the trait. The model can be considered as

$$y=1_{n}\mu +X\beta +u+\varepsilon ,$$

$$\beta ∼\pi N\left(0,\sigma_{a}^{2}\tau^{-1}\right)+\left(1-\pi \right)\partial_{0},$$

$$u∼\text{MVN}\left(0,\sigma_{b}^{2}\tau^{-1}K\right),$$

$$\varepsilon ∼\text{MVN}\left(0,\tau^{-1}I_{n}\right),$$

where *µ* represents the mean of the trait of interest, *β* represents a vector of fixed (sparse) effects, *u* is a vector of random effects, *ε* is a vector of errors, π represents the proportion of variants which have an additional effect (over the polygenic effect provided by the LMM component), *τ* ^−1^ represents the residual variance, *σ_a_* is the magnitude of the non‐zero fixed effects, *δ*_0_ is a point mass at zero, *σ_b_* is the magnitude of the random effects, *K* is a variance‐covariance matrix of genotypes, and *I_n_* is an identity matrix.

In practice, the model is reparameterised in terms of PVE (*ρ*), which is the proportion of variance explained by the sparse and random effects together, and PGE (*h*), which is the proportion of variance explained by only the sparse effects. *ρ* and *h*, in addition to *π*, are the model hyperparameters estimated through Markov chain Monte Carlo (MCMC). Here, BSLMM was implemented through GEMMA (Zhou et al., 2013). We used 1,000 burn‐in iterations and 10,000 MCMC iterations per gene for the determination of hyperparameters. In addition, we tested BSLMM using 10,000 burn‐in iterations and 100,000 MCMC iterations for 250 genes on chromosome 18. As we did not see an increase in predictive performance with the longer MCMC run (Figure S1), we chose to use the shorter MCMC run for the whole genome.

For each run of the BSLMM, we estimated how well the Markov chain for each hyperparameter mixed by calculating the autocorrelation between Markov chain states. For each chain, we defined good mixing as being achieved when autocorrelation at lag 100 was between −0.1 and 0.1. Chains showing autocorrelation values outside this range at lag 100 were considered to have mixed poorly.

#### 2.2.5 Best linear unbiased predictor

The BLUP can be derived from a random‐effects regression model

$$y_{i}=u_{i}+\varepsilon_{i},$$

where *y*_*i*_ represents the phenotype of individual *i*, *u*_*i*_ represents a random effect representing the overall genetic effect (summed over all loci) operating in individual *i* and *ε*_*i*_ is the residual term. These are assumed to follow a normal multivariate distribution:

$$\left[\begin{array}{c} u\\ \varepsilon \\ y \end{array}\right]∼\text{MVN}\left[0,\begin{array}{ccc} G\sigma_{u}^{2} & 0 & G\sigma_{u}^{2}\\ 0 & I\sigma_{\varepsilon }^{2} & I\sigma_{\varepsilon }^{2}\\ G\sigma_{u}^{2} & I\sigma_{\varepsilon }^{2} & G\sigma_{u}^{2}+I\sigma_{\varepsilon }^{2} \end{array}\right],$$

$$G=XX^{T}/p,$$

where ***G*** represents a genetic relationship matrix (GRM) defined using genotypes of all SNPs within 1 Mb of the gene start or end sites, *p* represents the number of SNPs used to generate the GRM, and ***I*** is an identity matrix. This model tends to produce a more polygenic solution where many SNPs have small effects on the phenotype of interest. Further details on the determination of marker effects and predictions can be found in the Supporting Information note of de Los Campos, Vazquez, Fernando, Klimentidis, and Sorensen, (2013). It was implemented here using GEMMA.

#### 2.2.6 Random Forests

Random Forests is a tree‐based machine learning method for classification and regression that was initially proposed by Breiman (2001). Random Forests typically use a standard algorithm. First, bagging is used to create many different training data sets from the initial data. Each of these data sets is then used to construct a decision tree. In the case of Random Forests, feature bagging is also performed, meaning for each tree a different random subset of the covariates is used to construct the tree. This procedure is performed for all the training data sets, resulting in a “forest” of decision trees. The average decision of the forest is then used as the prediction for the trait of interest. Random Forests were implemented using the R package *ranger*.

### 2.3 Assessing prediction performance

We used the Geuvadis data to examine gene expression prediction accuracy under a number of scenarios:

#### 2.3.1 10‐fold nested cross‐validation on EUR samples

To initially compare the performance of the seven different statistical methods, we used 10‐fold nested cross‐validation on the 373 EUR samples in Geuvadis. This consisted of one cross‐validation within another. These are termed the inner loop (used to tune model parameters) and the outer loop (used to evaluate model performance). The 373 samples were split into 10 groups of equal size. For a single fold of the outer loop, nine groups were combined into a single data set to be used for training a gene expression prediction model (training set), and the remaining one group was used to evaluate the performance of the model (test set).

The inner loop 10‐fold cross‐validation was then performed on the training set. This inner loop was used to tune any model parameters. For ridge regression, elastic net (*α* = .5) and LASSO, this inner loop was used to tune the *λ* parameter. For elastic net (*α* by cross‐validation), the inner loop was also used to tune the *α* parameter. In each case, the parameter value was chosen as that which maximized the correlation coefficient (*R*) between the predicted and the observed expression values, within this inner loop. BSLMM and BLUP did not have any parameters that could be tuned via cross‐validation, so this inner loop was not performed for these methods.

Once appropriate parameter values had been chosen using the inner loop, a gene expression prediction model was trained on the full training set using the appropriate parameter values. The resulting model was then applied to the test set, and expression was predicted for these samples. The correlation coefficient (*R*) between predicted expression and measured expression was then calculated in the test set. This gave a single *R‐*value for onefold of the outer loop.

This procedure was then repeated for each of the remaining ninefold of the outer loop, each time using a different 10th of the samples as the test set, so that each Geuvadis sample appeared in the test set once. This gave 10 *R* values—one for each fold of the outer loop. The prediction performance of a method for a gene was then defined as the mean of these 10 *R* measures.

This 10‐fold nested cross‐validation was performed seven times (once for each different method) for each gene with available gene expression data.

#### 2.3.2 Prediction at smaller sample sizes

To examine the effect of sample size on gene expression prediction accuracy, we repeated the 10‐fold nested cross‐validation procedure described above, using the 373 EUR Geuvadis samples. However, in each fold of the outer loop of cross‐validation, we used 1 of the 10 groups as the training set, and the remaining nine groups combined as the test set. The rest of the procedure was unchanged. *R* estimates obtained using this nested cross‐validation with the reduced training set were then compared with *R* estimates from our original cross‐validation.

#### 2.3.3 Prediction into a different population

To evaluate the predictive ability of models trained using samples of one ancestry to predict the expression of samples of an alternative ancestry, we used the 89 YRI samples. Initially, gene expression prediction models were trained using the whole set of 373 EUR samples, with model parameters tuned via 10‐fold cross‐validation within the EUR samples. These prediction models were then applied to the 89 YRI samples, and predictive performance was evaluated as the correlation (*R*) between gene expression predicted by the EUR‐trained models and measured gene expression. The analysis was then repeated using 90% of EUR samples to train models, and 37 YRI samples to test models, giving training and testing set sample sizes equivalent to those used for onefold of the 10‐fold nested cross‐validation on EUR samples.

To perform the reverse analysis, models were trained on the 89 YRI samples, with parameters tuned via 10‐fold cross‐validation. These models were then applied to the 373 EUR samples, and R between gene expression predicted by the YRI models and measured EUR expression was calculated. In addition, 10‐fold nested cross‐validation was performed on the 89 YRI samples using the same procedure as used for the 10‐fold nested cross‐validation on EUR samples. The *R* estimates from the application of YRI models to EUR samples were compared with the *R* estimates obtained from the 10‐fold nested cross‐validation on the 89 YRI samples.

Finally, a combined analysis was performed. The 373 EUR and 89 YRI samples were combined into a single group of 462 samples, and down‐sampled to 373 samples, keeping the relative proportion of EUR and YRI samples the same as in the larger group of 462 samples. 10‐fold nested cross‐validation was performed on this mixed group of 373 samples, using the procedure as described above. The *R* estimates obtained from this were then compared with *R* estimates obtained from the 10‐fold nested cross‐validation on the 373 EUR samples.

### 2.4 Gene set enrichment

Gene set enrichment was conducted using FUMA (Watanabe, Taskesen, van Bochoven, & Posthuma, 2017). A list of genes that achieved *R* ≥ 0.5 (for any of the seven methods tested) in the 10‐fold nested cross‐validation using the 373 EUR Geuvadis samples were used as the input for the “gene2func” option. The MHC region was not excluded.

### 2.5 Estimation of gene expression heritability

For each gene, the heritability of its expression attributable to SNPs within 1 Mb of the gene start or end site was calculated using restricted maximum likelihood (REML) analysis implemented in GCTA (Yang, Lee, Goddard, & Visscher, 2011). For each gene, all SNPs within 1 Mb of the gene were used to construct a GRM. The proportion of the variance of gene expression explained by these SNPs (the narrow‐sense heritability) was then estimated using REML analysis in GCTA. As the intention was to compare heritability estimates with prediction accuracy estimates from 10‐fold cross‐validation, the heritability estimates from REML were restricted to fall within the [0,1] range.

### 2.6 Application to WTCCC1 data

Using each of the seven methods, gene expression prediction models were trained using data from all 373 EUR samples in Geuvadis. To avoid issues caused by SNPs in the prediction models not being present in the GWAS data, these prediction models were trained using only the set of SNPs that were present in the WTCCC1 T1D data. Each of these prediction models was then applied to individual‐level imputed genotype dosages of T1D cases and controls from WTCCC1 to impute their expression. Imputed expression was then tested for association with T1D status using logistic regression, with no additional covariates included in the regression model. The association statistics for genes successfully modeled by all seven approaches were then compared.

### 2.7 Comparison with PrediXcan models

Gene expression prediction models for 48 different GTEx tissues from the PrediXcan software package were downloaded from predictdb.org. These prediction models had been trained by the developers of the PrediXcan software packages using elastic net (with *α* set to .5). Each set of prediction models was applied to genotype data for the 373 EUR samples in Geuvadis, and for each of these prediction models, the correlation coefficient (*R*) between measured Geuvadis expression and expression predicted using the GTEx‐informed model was calculated.

## 3 RESULTS

### 3.1 Comparison of statistical modeling approaches for gene expression prediction

We first aimed to compare the performance of seven different statistical approaches for the prediction of gene expression from SNP genotypes. The chosen modeling approaches were: ridge regression, elastic net (with the *α* parameter set to .5), elastic net (with *α* determined by cross‐validation), LASSO, BSLMM, BLUP, and Random Forests. To compare the approaches, LCL expression levels of 23,722 genes from the Geuvadis project were used. For each of the seven approaches, 10‐fold nested cross‐validation was performed on data from the 373 Geuvadis samples of EUR origin. In each of the 10 folds, 90% of samples (the training set) were used to train a model, which was applied to the remaining 10% (the test set). Correlation (*R*) between the predicted and measured expression of each gene in the test set was calculated in each fold. For each gene, we defined prediction accuracy as the mean of the 10*R* estimates.

In total, the expression of 22,218 genes could be predicted by all seven methods. Overall, the BSLMM showed the best average prediction performance (average mean *R* = 0.0743) across these 22,218 genes (Table 1). Behind the BSLMM, the other methods performed similarly to each other, with the Random Forests and the sparse penalized regression methods slightly outperforming the polygenic methods, on average. While BSLMM achieved the highest average *R* across the 22,218 genes, examination of its MCMC trace plots revealed a consistent failure to converge for some of its hyperparameters (Figure S2). In total, we estimated that 208,702 of the 225,170 BSLMM models fitted during the 10‐fold cross‐validation showed poor MCMC mixing (indicative of poor convergence) for at least one of the hyperparameters, raising concerns about its use for prediction of gene expression.

**Table 1:** Mean *R* estimates across 22,218 genes from 10‐fold nested cross‐validation using seven different statistical methods

Method	Mean *R* (across 22,218 genes)
Ridge regression	0.0587
Elastic net (*α* = .5)	0.0634
Elastic net (*α* tuned by cross‐validation)	0.0656
LASSO	0.0626
BSLMM	0.0743
BLUP	0.0608
Random Forests	0.0641

Abbreviations: BSLMM, Bayesian sparse linear mixed model; BLUP, best linear unbiased predictor; EUR, European; LASSO, least absolute angle and selection operator.

**Figure 1:**
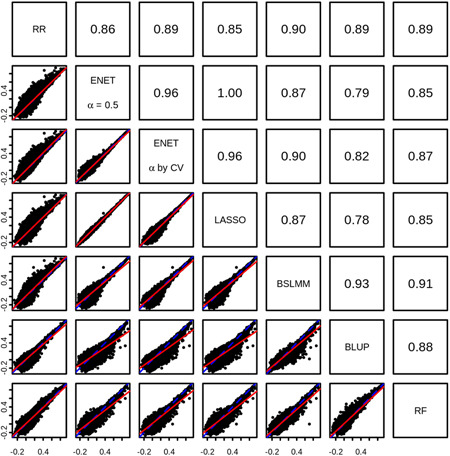
Correlation between *R* estimates from seven different modeling approaches. In the lower panels, each point represents a single gene, and the *R* estimate obtained from the two corresponding methods are shown on the *x* and *y* axes. Also shown are the line of equality (blue‐dashed line) and the best fit line between *x* and *y* (red solid line). In the upper panels, the Pearson correlation between *R* estimates from pairs of methods are shown. Overall, *R* estimates from all methods were highly correlated, with all pairwise correlations ≥0.78

We observed that *R* estimates from the seven methods were all highly correlated with one another (Figure 1), indicating that genes predicted well with one method were also predicted well with others. Despite this, some genes showed a large difference in the *R* estimates achieved by different methods. For example, *HSPA12B* showed *R* = 0.877 with elastic net (*α* = .5) and *R* = .885 with LASSO, but only achieved *R* = .425 with ridge regression and *R* = .332 with BLUP. In total, for 108 genes we observed a difference in *R* of at least 0.3 between any two methods. In 94 of these cases, a method with assumptions of sparsity showed the greater *R*, while a more polygenic method showed the lower *R*. For these genes, there was a clear benefit in using the more sparse method.

**Figure 2:**
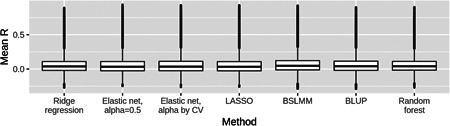
Boxplots of gene expression prediction accuracy estimates from seven methods. Each boxplot shows the distribution of *R* estimates (between predicted and observed expression) for 22,218 genes from 10‐fold nested cross‐validation for one statistical method. The central line within the box represents the median, with the upper and lower quartiles shown as the hinges. Each boxplot is heavily skewed towards zero, indicating that for most genes, prediction accuracy was poor. BSLMM, Bayesian sparse linear mixed model; BLUP, best linear unbiased predictor, LASSO, least absolute angle and selection operator

**Figure 3:**
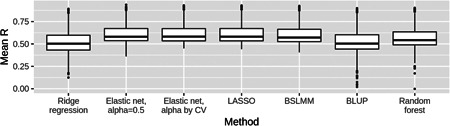
Boxplots of gene expression prediction accuracy estimates from seven methods for well‐predicted genes. Each boxplot shows the distribution of *R* estimates (between predicted and observed expression) for 480 genes from 10‐fold nested cross‐validation for one statistical method. These genes had *R* ≥ 0.5 from at least one of the seven methods. The central line within the box represents the median, with the upper and lower quartiles shown as the hinges. Boxplots for the methods with assumptions of sparsity (LASSO, Elastic net, and BSLMM) show greater medians than plots for the more polygenic methods, and are less skewed toward 0, indicating better performance. BSLMM, Bayesian sparse linear mi xed model; BLUP, best linear unbiased predictor, LASSO, least absolute angle and selection operator

When examining results on a gene‐by‐gene basis, it is clear that the expression of most genes could not be accurately predicted from local SNP genotypes by any of the seven methods tested here. Distributions of *R* estimates for the 22,218 genes were heavily skewed toward zero for all seven methods (Figure 2), with negative correlations between predicted and measured expression observed for some genes. However, each distribution showed a trail of points corresponding to genes with high *R* estimates, indicating that the expression of some genes could be well predicted from local SNP genotypes. A total of 480 genes (2.16% of all genes tested) showed *R* ≥ 0.5 from any of the seven methods, and are subsequently defined as “well‐predicted” genes. When considering only these genes, the modeling methods with assumptions of sparsity outperformed the more polygenic methods even more strongly than observed previously (Figure 3). Gene set enrichment analysis on these genes found enrichment of seven Gene Ontology gene sets and four GWAS catalog gene sets (Table S1), mostly related to immunity. This probably reflects the immune nature of LCLs in which Geuvadis expression was measured, and is likely driven by the presence of a number of HLA genes in this list, including *HLA‐DRB1*, *HLA‐DRB5*, and *HLA‐DQA1*.

The upper bound of prediction accuracy achievable here is given by the heritability of gene expression attributable to local SNPs. To identify this upper bound for each gene, we next estimated the heritability of each transcript that was attributable to the SNPs within 1 Mb of the gene using GCTA. Overall, estimates of local heritability were highly correlated (*r* = .85) and (with a small number of exceptions) showed strong concordance with estimates of prediction accuracy obtained using elastic net (*α* = .5; Figure S3). On average, heritability estimates were slightly larger than prediction accuracy estimates, indicating that there was some potential room for improvement to prediction accuracy.

### 3.2 Comparison of statistical modeling approaches for TWAS

We next sought to examine how the seven methods compared in the context of a TWAS, where prediction models are applied to GWAS data to identify gene expression—trait associations. To do this, gene expression prediction models were trained on the full set of 373 EUR Geuvadis samples using each of the seven methods and were applied to type 1 diabetes (T1D) GWAS data from WTCCC1.

**Figure 4:**
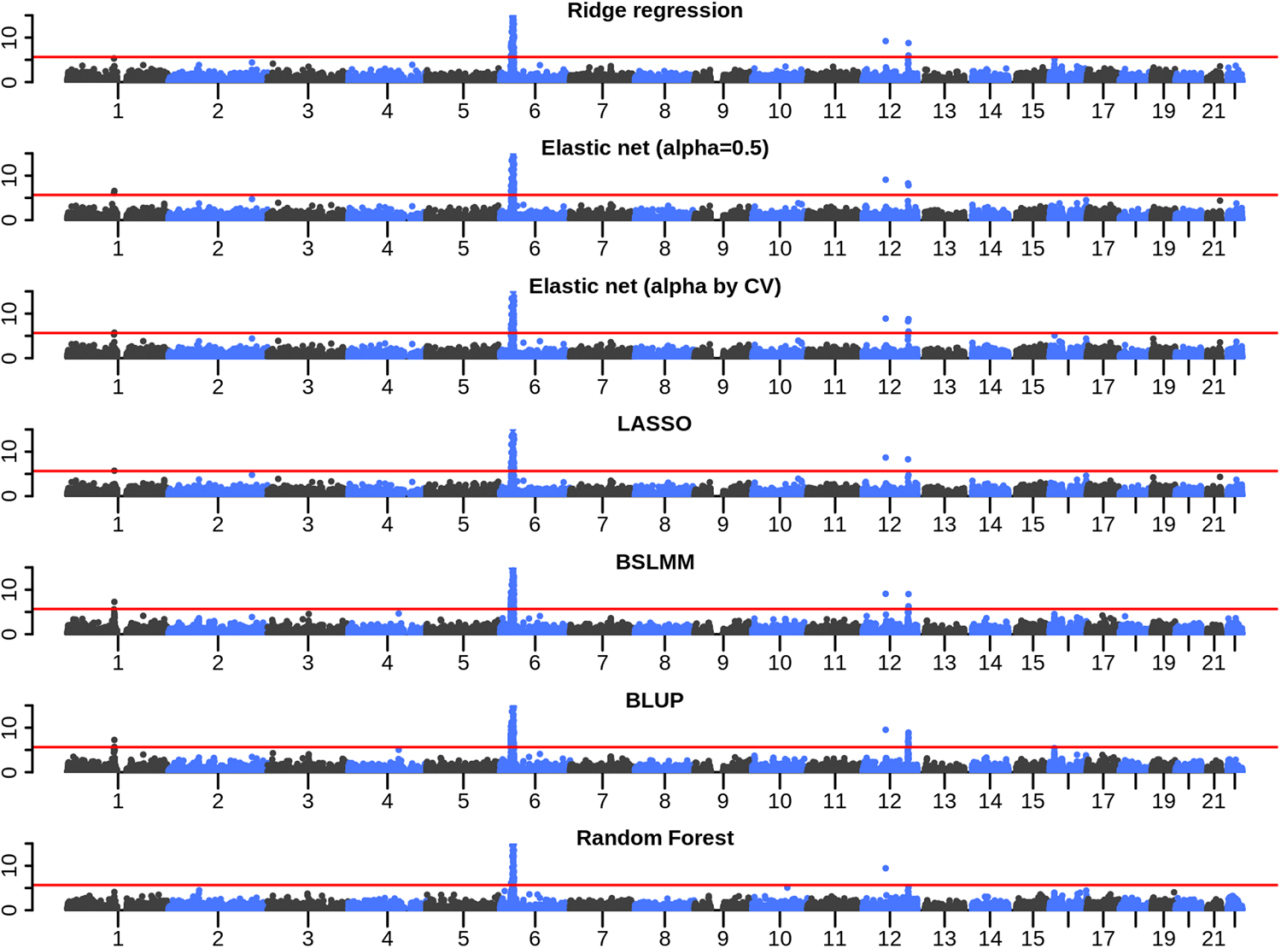
Manhattan plots from the application of gene expression prediction models to WTCCC T1D GWAS data. Each plot shows the results of a TWAS on WTCCC T1D data using gene expression prediction models trained with a different statistical method. In each plot, each point represents a gene, plotted by its genomic position (defined by the TSS) on the *x* axis, and its *p* value in the TWAS on the *y* axis. The red lines indicate Bonferroni‐corrected significance thresholds. BSLMM, Bayesian sparse linear mixed model; BLUP, best linear unbiased predictor, LASSO, least absolute angle and selection operator

**Figure 5:**
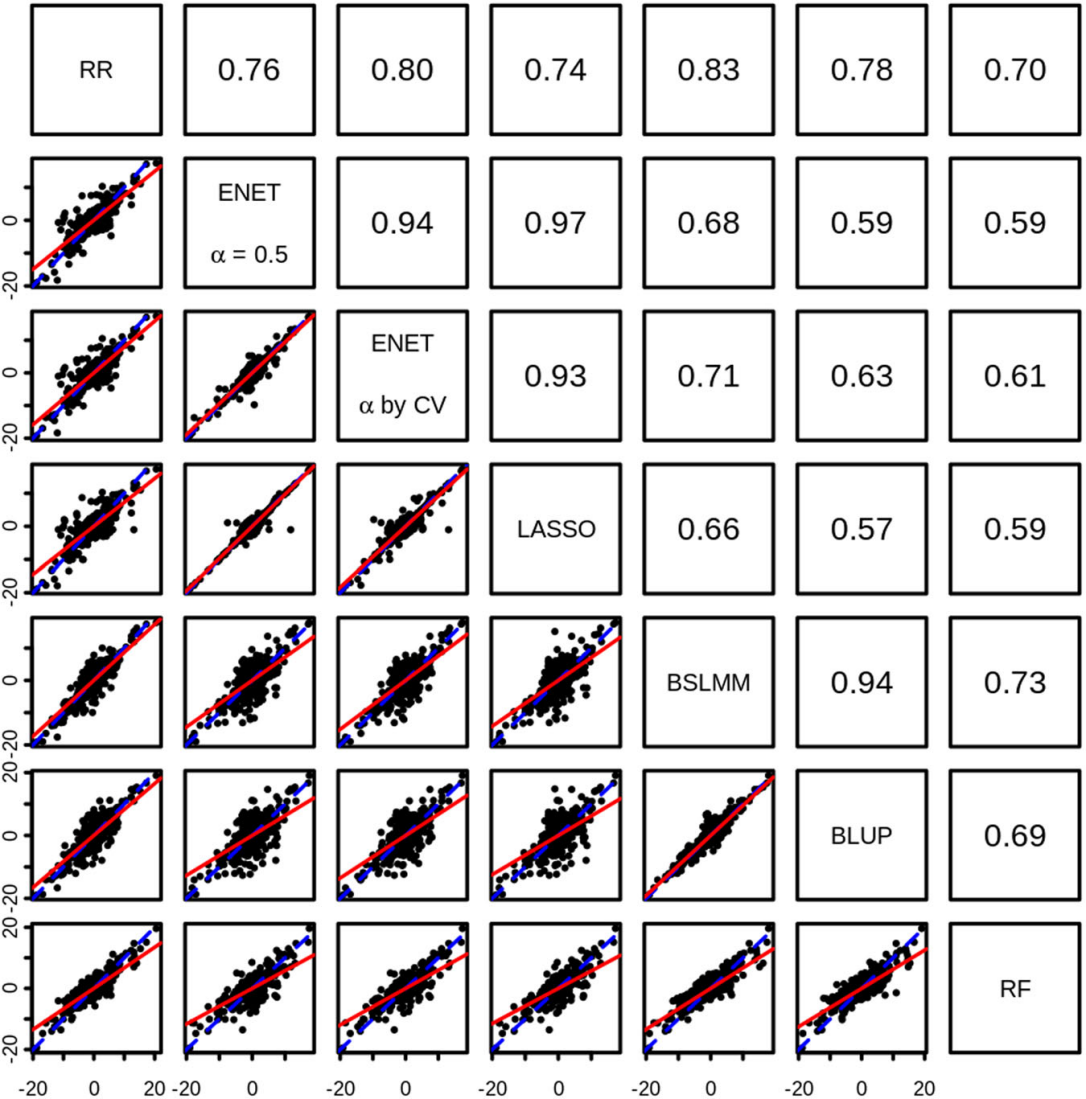
Correlation between *z* scores from TWAS on WTCCC T1D data using seven different modeling approaches. In lower panels, each point shows a single gene, with the *z* scores from TWAS using models trained with two different statistical approaches shown on the *x* and *y* axes. Also shown are the line of equality (blue‐dashed line) and the best fit line between *x* and *y* (red solid line). Upper panels show Pearson correlation estimates between *z* scores from TWAS using the two corresponding methods. *Z* scores from the seven approaches were highly correlated

Overall, all seven methods tended to find associations in similar genomic regions (Figure 4). All methods identified associations with a number of genes in the MHC region on chromosome 6, and a number of associations on chromosome 12 at 12q13 and 12q24. Both of these regions were also identified through traditional GWAS of the T1D WTCCC data. For the genes tested by all seven approaches, *z* scores were highly correlated (Figure 5), with no approach showing greater average *z* scores than another approach.

### 3.3 Investigation of the impact of sample size in gene expression prediction

A factor known to affect the accuracy of the prediction of complex traits is the sample size of the data set used to train prediction models. To investigate the effect of sample size on gene expression prediction accuracy, we repeated the 10‐fold nested cross‐validation, but using 10% of samples as the training set and the remaining 90% as the test set in each fold. Estimates of prediction accuracy achieved with this reduced training set were then compared with those previously obtained from the 10‐fold nested cross‐validation using the larger training set.

**Figure 6:**
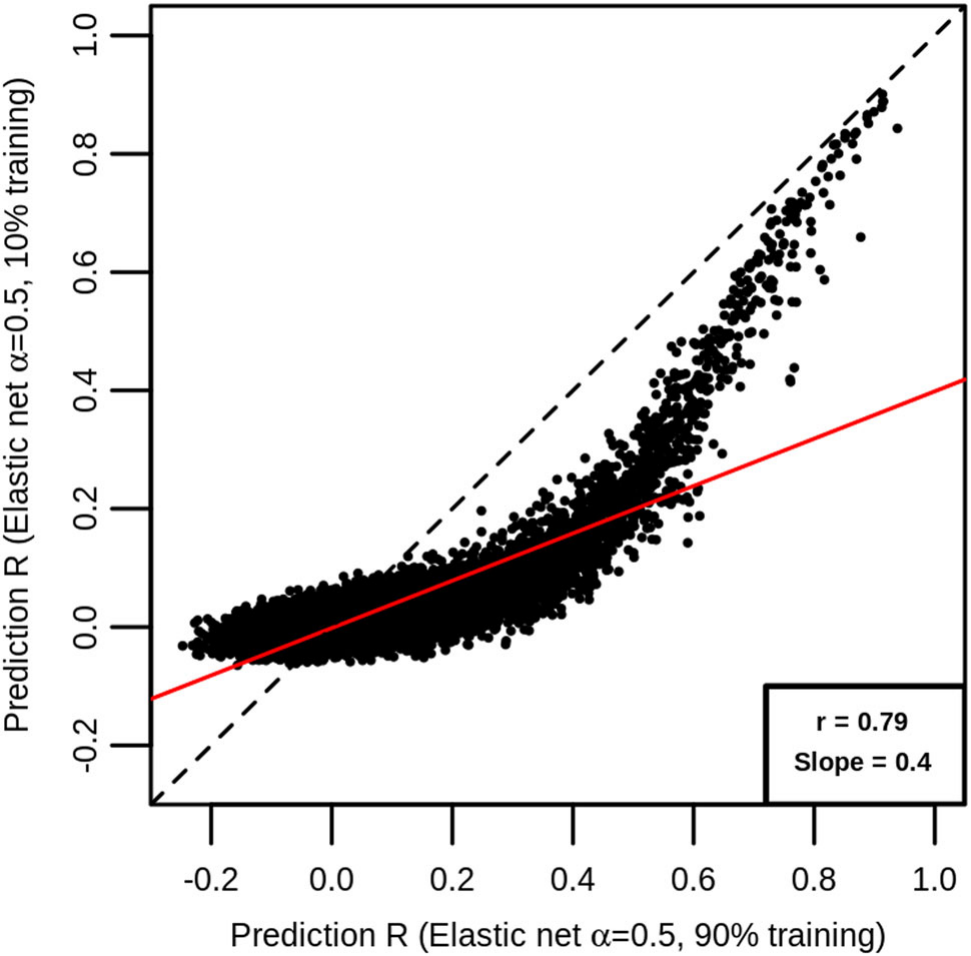
Comparison between prediction accuracy estimates at large and small sample sizes. Each point represents a gene, with its *R* estimate from 10‐fold nested cross‐validation using 90% of EUR samples as the model training set on the *x* axis, and the *R* estimate from 10‐fold nested cross‐validation using 10% of EUR samples as the model training set on the *y* axis. All *R* estimates were obtained using the elastic net with *α* = .5. Also shown are the line of equality (black dashed) and a line of best fit (red solid), with the correlation between *x* and *y* and the slope of the best fit line shown in the bottom right corner. Most points lie below the line of equality, and the slope of the best fit line is below 1, indicating that average performance was greater when using the larger sample size. EUR, European

Overall, the average prediction accuracy was reduced when using the smaller training set. For elastic net (*α* = .5), 22,490 genes had predictions from both the analyses using the larger and the reduced training sets. Of these, 14,019 (62.3%) showed a smaller *R* in the analysis using the reduced training set (Figure 6). We repeated this analysis using the other six statistical methods tested earlier, and in each instance, we observed poorer prediction accuracy in the analysis with the reduced training set (Table 2). Prediction accuracy estimates from the analyses using the larger and reduced sample sizes were, however, highly correlated (*r* = .79), and interestingly, many genes that achieved large R estimates in the 90% training set analysis also achieved large *R* estimates at the reduced sample size. For example, *RPS26* showed *R* = 0.913 in the larger training set analysis and *R* = 0.888 in the reduced training set analysis. Similarly, *AC008957.1* showed *R* = 0.915 in the analysis using the larger training set, and *R* = 0.889 in the reduced training set analysis. Thus, it seems that for genes where the relationship between expression and local SNPs is strong enough, even small sample sizes are sufficient for constructing models that predict expression well.

**Table 2:** Mean *R* estimates obtained using 10‐fold nested cross‐validation with large and small model training set sample sizes using seven different statistical approaches

Method	Number of genes	Mean *R* (90% training set)	Mean *R* (10% training set)
Ridge regression	22,490	0.0576	0.0256
Elastic net (*α* = .5)	22,490	0.0625	0.0234
Elastic net (*α* tuned by cross‐validation)	22,490	0.0646	0.0246
LASSO	22,490	0.0617	0.0213
BSLMM	22,509	0.0723	0.0303
BLUP	22,222	0.0608	0.0248
Random Forests	22,498	0.0628	0.0286

Abbreviations: BSLMM, Bayesian sparse linear mixed model; BLUP, best linear unbiased predictor; EUR, European; LASSO, least absolute angle and selection operator.

To further investigate the effect of sample size, 10‐fold nested cross‐validation was repeated a further seven times, using 20%, 30%, 40%, 50%, 60%, 70%, and 80% of samples as the training set in each analysis. Across the genes modeled at all sample sizes, we observed a clear improvement on average prediction accuracy with each increase in the model training set sample size (Figure S4A). This relationship did not plateau at the largest available sample size, indicating that a further increase in sample size may boost the average prediction accuracy observed here. Despite this, a plateau in prediction accuracy was observed for some genes, with no further increase beyond a given sample size (Figure S4B), indicating that the upper limit of prediction accuracy imposed by the local heritability of expression at these genes had been reached at the sample sizes used here.

### 3.4 Investigation of the impact of ancestry in gene expression prediction

We next sought to investigate how well prediction models trained using samples of one ancestry were able to predict the gene expression of samples of different ancestry. To do this, prediction models were trained on the 373 EUR Geuvadis samples using elastic net (with *α* = .5) and applied to genotype data for 89 YRI Geuvadis samples, and the correlation (*R*) between predicted expression and measured expression was calculated. These *R* measures were then compared with the *R* achieved by elastic net (with *α* = .5) from 10‐fold nested cross‐validation within the EUR samples.

**Figure 7:**
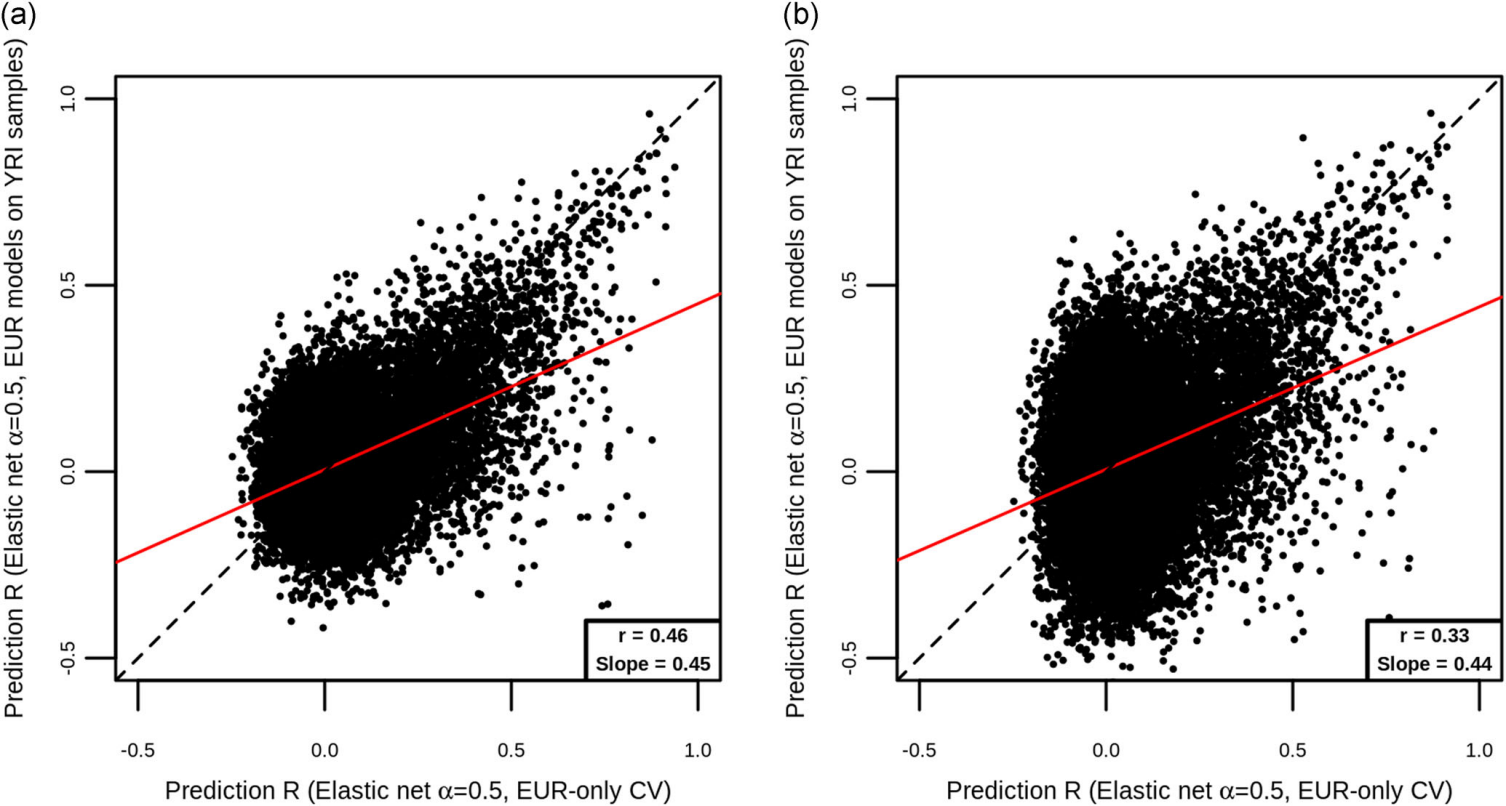
Comparison of prediction accuracy estimates when EUR‐trained models are applied to EUR and YRI populations. On both plots, each point represents a gene, and shown are the *R* estimate from 10‐fold nested cross‐validation within EUR samples (*x* axis), and the *R* between expression predicted using models trained on EUR samples and applied to YRI samples, and measured YRI expression (*y* axis). Plot (a) corresponds to the analysis where sample sizes of the model training and testing sets used for the within‐EUR analysis were not the same as the sample sizes used in the across‐ancestries analysis. Plot (b) corresponds to the analysis where the sample sizes of the model training and testing sets were the same in both the within‐EUR and across‐ancestries analyses. Also shown are the line of equality (black dashed) and a line of best fit (red solid), with the correlation between *x* and *y* and the slope of the best fit line shown in the bottom right corner. Most points lie below the line of equality, and the slope of the best fit line is below 1, indicating that prediction models trained on EUR samples perform better at predicting EUR expression than YRI expression; EUR, European; YRI, Yoruba

Overall, gene expression prediction models trained in a population of one ancestry tended to perform less well at predicting the expression of samples of a different ancestry (Figure 7a). Of the 22,493 genes for which we had *R* measures both from the application of EUR models to YRI samples and also from 10‐fold nested cross‐validation within EUR samples, 12,828 (57.0%) showed larger *R* from 10‐fold nested cross‐validation within EUR samples. Across the 22,493 genes, the average prediction accuracy obtained from 10‐fold cross‐validation on EUR samples (mean *R* = 0.0625) was greater than the average accuracy from the application of EUR models to YRI samples (mean *R* = 0.0332). However, there was no consistent pattern, with some genes showing greater *R* from the application of EUR models to YRI samples. We repeated this analysis with the other six statistical methods and consistently observed poorer average prediction when predicting into YRI samples (Table 3).

**Table 3:** Mean *R* estimates obtained from 10‐fold nested cross‐validation within EUR samples and from the application of EUR‐trained models to YRI samples using seven different statistical approaches

Method	Number of genes	Mean *R* (from within EUR cross‐validation)	Mean *R* (from application of EUR‐trained models to YRI samples)
Ridge regression	21,891	0.0602	0.0274
Elastic net (*α* = .5)	22,493	0.0625	0.0332
Elastic net (*α* tuned by cross‐validation)	22,363	0.0651	0.0351
LASSO	22,492	0.0617	0.0320
BSLMM	22,498	0.0724	0.0395
BLUP	21,627	0.0635	0.0313
Random Forests	22,498	0.0628	0.0303

Abbreviations: BSLMM, Bayesian sparse linear mixed model; BLUP, best linear unbiased predictor; EUR, European; LASSO, least absolute angle and selection operator.

To account for the differences in sample size in the above analysis compared to our previous investigation in European samples, we retrained prediction models on 90% of the EUR Geuvadis samples and applied them to 37 YRI samples, matching the training and test set sample sizes used for onefold of the 10‐fold nested cross‐validation on EUR samples. Again, we observed that prediction models trained in a population of one ancestry performed more poorly when applied to samples of different ancestry (Figure 7b).

We next performed the reverse of this analysis by training models on the 89 YRI samples using elastic net (with *α* = .5) and applying them to the EUR samples, and calculating *R* between predicted and measured expression in the EUR samples. We also performed 10‐fold nested cross‐validation using only the 89 YRI samples and then compared the *R* estimates from this YRI‐only 10‐fold nested cross‐validation with *R* estimates from the application of YRI‐trained models to EUR samples. Across the 22,334 genes, the average prediction accuracy from 10‐fold cross‐validation on YRI samples (mean *R* = 0.0317) was greater than the average accuracy from the application of YRI models to EUR samples (mean *R* = 0.0184; Figure S5A), reinforcing our earlier findings. As above, to account for sample size differences, we repeated the analysis by training models on 90% of YRI Geuvadis and applying them to nine EUR samples, matching the sample sizes to onefold of the 10‐fold cross‐validation on the 89 YRI samples. While *R* estimates showed much variation, likely reflecting the smaller sample sizes used for both prediction and testing, we again observed that average prediction accuracy was poorer when predicting across populations (Figure S5B).

Finally, we combined the EUR and YRI samples into a single group of 462 samples. We then extracted 373 samples, keeping the proportion of EUR and YRI samples the same as in the larger group of 462 samples. Using this mixture of EUR and YRI samples, we performed 10‐fold nested cross‐validation on this group using an elastic net (with *α* = .5). *R* values achieved in this mixed sample were highly correlated (*r* = .780) with those achieved from 10‐fold nested cross‐validation using only the 373 EUR samples (Figure S6). Across the 22,498 genes, the average prediction accuracy estimate obtained from this mixed sample 10‐fold cross‐validation (mean *R* = 0.0609) was similar to the average prediction accuracy estimate obtained from 10‐fold cross‐validation on EUR samples (mean *R* = 0.0625), demonstrating that even when the population contains samples from different ancestries, the prediction accuracy mimics that achieved when using a single ancestry, as long as the composition of the training population matches that of the testing population.

### 3.5 Application of GTEx‐trained models to Geuvadis data

Up to this point, our analyses have relied on splitting Geuvadis data into different subsets, using models trained in one subset to predict expression in another. However, these subsets of Geuvadis data have all undergone data collection and processing in the same manner, and thus are likely to be very similar. This does not reflect the most likely real‐life use of TWAS, in which the data sets used for model training and for application may be very different. To investigate a more realistic scenario, prediction models trained using GTEx EBV‐transformed LCL expression data (taken from the PrediXcan software package) were applied to Geuvadis EUR samples. Correlation between expression predicted with GTEx‐trained models and measured Geuvadis expression was calculated.

**Figure 8:**
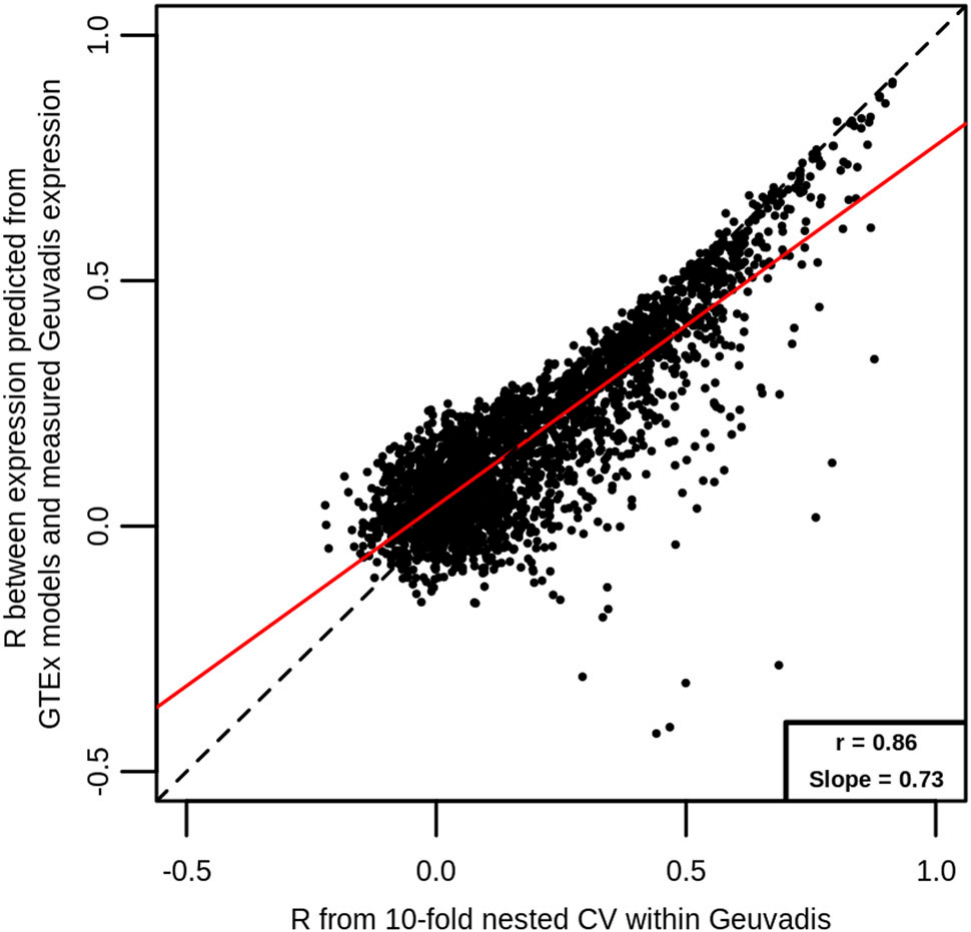
Comparison of Geuvadis‐trained models and GTEx‐trained models at predicting Geuvadis expression. Each point represents a gene, and shown are the *R* estimates from 10‐fold nested cross‐validation within EUR samples on Geuvadis data (*x* axis), and *R* between measured Geuvadis expression and expression predicted using GTEx‐trained models. Also shown are the line of equality (black dashed) and a line of best fit (red solid), with the correlation between *x* and *y* and the slope of the best fit line shown in the bottom right corner. As most points lie below the line of equality and the slope of the best fit line is below 1, this implies that Geuvadis‐trained models are better able to predict Geuvadis expression than GTEx‐trained models

Overall, prediction accuracy estimates from using the PrediXcan GTEx‐trained models were very similar to those achieved from 10‐fold nested cross‐validation on Geuvadis data using elastic net (*α* = .5; Figure 8). In total, there were 2,737 genes for which a GTEx‐trained prediction model and measured expression data in Geuvadis were available. Genes predicted well using 10‐fold nested cross‐validation were also well predicted using GTEx‐trained models, including *RPS26*, which achieved *R* = 0.905 from the application of the GTEx model to Geuvadis, and *R* = 0.913 from 10‐fold nested cross‐validation within Geuvadis. Overall, *R* estimates from 10‐fold nested cross‐validation and from GTEx‐trained models were highly correlated (*r* = .86), although those from GTEx‐trained models tended to be slightly smaller.

### 3.6 Investigation of prediction across different tissues

We next sought to examine how models trained using GTEx expression data from a range of tissues performed at the prediction of Geuvadis LCL gene expression. To do this, 47 sets of gene expression prediction models taken from the PrediXcan software package, each trained using GTEx expression data from a tissue other than LCLs (in which Geuvadis expression was measured), were applied to Geuvadis data and the correlation between predicted and measured expression was calculated. For each of the 47 GTEx non‐LCL tissues, the average level of prediction accuracy achieved (across gene‐specific prediction models) was lower than that achieved (average *R* = 0.188) with the GTEx LCL models (Table S2).

We then directly compared the accuracy of predicted expression from models trained in each of the 47 GTEx non‐LCL tissues with the accuracy of predicted expression obtained from models trained in the GTEx LCL data, using only genes for which there was a prediction model available in both GTEx LCL and in the non‐LCL GTEx tissue of interest. When examining these results on a gene‐by‐gene basis, we observed that for many genes the non‐LCL models could predict Geuvadis expression with similar accuracy to LCL models, yet there was a group of genes for which the LCL model clearly outperformed the non‐LCL model (Figure S7). This resulted in the non‐LCL prediction models achieving slightly poorer prediction accuracy than the LCL prediction models on average.

In total, 53.3% of prediction accuracy estimates achieved by non‐LCL prediction models were within 0.05 of the prediction accuracy estimate achieved by the LCL model for the same gene, indicating similar performance and potentially shared regulation across different tissues. One gene for which non‐LCL prediction models achieved similar prediction accuracy to the LCL model was *RPS26*. Prediction models for this gene were available for all 48 GTEx tissues, and prediction models for all tissues achieved *R* ≥ 0.8 when applied to the Geuvadis data (Figure S8). In contrast, we found that 10.3% of prediction accuracy estimates achieved by non‐LCL prediction models were at least 0.2 less than that achieved by the LCL model for the same gene. One example of this was *NDUFAF1*, for which the GTEx LCL model achieved a prediction accuracy estimate of 0.686, whereas the GTEx transverse colon model achieved an estimate of 0.128. In instances such as this, the LCL models clearly outperformed the non‐LCL models.

## 4 DISCUSSION

In recent years, TWAS has become a popular post‐GWAS approach for integrating genotype and expression data to identify genes potentially underlying phenotypes of interest. The power of TWAS to detect true gene expression‐phenotype associations relies partially on accurate imputation of gene expression values. Accuracy of imputation is dependent on a number of factors, including (but not limited to) the statistical method used to construct the prediction model, sample size, ancestry matching, and tissue matching. Here, we examined how each of these factors affects the accuracy of gene expression prediction, and thus how they may impact power in TWAS.

By using cross‐validation to train and test prediction models, we found that statistical models that assumed sparsity predicted gene expression levels slightly better than those that assumed polygenicity, corroborating similar findings from previous studies (Zeng et al., 2017). This was especially the case for genes where expression could be predicted well from SNP genotypes. As prediction models were constructed using only SNPs local to each gene, and the local genetic architecture of gene expression is thought to be sparse (Wheeler et al., 2016), this result is perhaps unsurprising. As there are thought to be many weak trans‐eQTLs (expression quantitative trait loci) acting on gene expression (indicative of a more polygenic distal genetic architecture; Liu, Li, & Pritchard, 2019), it is possible that the more polygenic methods may perform better if distal SNPs were also to be used for the construction of prediction models.

Here, we found that the best performing method was the BSLMM, which showed a slightly higher average prediction accuracy than Random Forests, elastic net, and LASSO. All the major transcriptome imputation software packages, including PrediXcan, S‐PrediXcan, and FUSION, construct their prediction models using elastic net with the *α* parameter set to .5. There thus could potentially be a slight gain on average prediction accuracy by either tuning the α parameter of the elastic net, or switching to Random Forests or the BSLMM. However, we found convergence issues when using the BSLMM, implying that the parameters used by BSLMM may not be correctly estimated from the data and raising concerns about the use of this method for modeling.

An important (and perhaps under‐appreciated) observation from our study is that the average prediction accuracy across the ~22,000 genes examined was typically very low, with many genes showing a cross‐validation *R* near 0. This was briefly shown in previous work (Fryett, Inshaw, Morris, & Cordell, 2018; Gamazon et al., 2015; Wheeler et al., 2016), but has not been explored in detail until now. Notably, we found that prediction accuracy estimates were concordant with, although on average slightly smaller than, estimates of local gene expression heritability, indicating that prediction models performed nearly as well as could be expected given the natural limit on prediction imposed by heritability. For genes with low *R*, we may expect TWAS power to be low. However, the power to detect associations through TWAS relies not only on the accuracy of gene expression imputation but also on the sample size of the GWAS data to be used. Thus, an association can still be detected for genes with low prediction *R*, provided a GWAS with a sufficiently large sample size is used. This is becoming more feasible with the advent of UK Biobank and other large population‐based resources.

We examined the effect of the sample size of the model training data set on expression prediction accuracy, finding that a reduced sample size led to poorer prediction. This has been observed in the context of prediction of other complex traits (Guo et al., 2016; Wei et al., 2013), and underlines the need for larger reference sample sets with measured genotype and expression. Current software packages for TWAS mainly use GTEx as a resource for the reference panel. While GTEx has measured genotype and expression across a range of tissues, allowing the development of tissue‐specific gene expression prediction models, for most tissues the sample size available in GTEx is small. An increase in sample size could improve prediction accuracy, and potentially subsequent TWAS power. A further reason to continue increasing sample size is that it will eventually allow for the inclusion of trans‐effects on expression. Current sample sizes are prohibitive for modelling the effects of all SNPs across the genome on gene expression, meaning that effects of SNPs distal to the gene on expression are missed. Many genes are known to have trans‐eQTLs (Brynedal et al., 2017), and trans‐eQTLs are thought to drive a number of disease associations (Kirsten et al., 2015; Westra et al., 2013). Thus, increasing sample size to the point where trans‐effects can be modelled may lead to an increase in prediction accuracy and TWAS power. However, inclusion of trans‐effects in prediction models also increases the likelihood of coprediction of multiple genes by a single prediction model, as trans‐eQTLs often regulate multiple genes (Brynedal et al., 2017). This could lead to difficulty in interpreting TWAS results. Further studies will be required to avoid this.

We also examined prediction across ancestries and observed a reduction on average prediction accuracy when predicting expression for a population of a different ancestry from the ancestry of the model training data. This may be due to differences in linkage disequilibrium across populations, differences in data processing between the samples from different populations, differences in the set of SNPs used for prediction, or differences in allele frequencies between populations as suggested in Mogil et al. (2018). Regardless of the reason, this would most likely reduce power in a subsequent TWAS. Most publicly available resources with measured genotypes and expression, including GTEx, are primarily made up of samples of European descent. Using these resources may lead to inaccurate expression prediction in populations of non‐European ancestry, and the detection of spurious associations through TWAS. While some effort has been made to generate gene expression prediction models in non‐European populations (Mogil et al., 2018), more population‐specific reference panels (especially with expression data for tissues other than blood) will need to be developed to allow accurate TWAS in populations of non‐European ancestry.

We also compared predictions from our Geuvadis‐trained models with those made by PrediXcan models. As expected, models trained using Geuvadis data slightly outperformed PrediXcan models at the prediction of Geuvadis data. This may reflect slight differences in data processing or population structure between the Geuvadis data and the GTEx data that PrediXcan models were trained in. This implies that training prediction models using data as similar as possible to the intended “test” data would likely result in more accurate predictions. While this may not be feasible for many software users, for large consortia where a proportion of samples have expression measures gathered, this may be a useful option. However, in uses such as this, it would still be important to consider the trade‐off between using similar data and using a larger sample size (which may be achieved by using a standard reference panel such as GTEx).

Next, we examined the issue of prediction across tissues. By applying GTEx‐trained models for non‐LCL tissues to Geuvadis data, we found that average prediction accuracy was reduced when the tissues of the model training data set and the model testing data set were different. We found that for many genes, models trained in non‐LCL tissues were able to predict LCL gene expression with similar accuracy to models trained using LCL expression data. Given the evidence of strong sharing of eQTLs across many tissues (GTEx Consortium, 2015), this is not surprising, but is reassuring, as it suggests that by using one tissue as a proxy for another, reasonable TWAS power may still be achieved for many genes. Despite this, there existed a set of genes for which the LCL‐trained models clearly outperformed models trained in other non‐LCL tissues, potentially reflecting tissue‐specific regulation of expression. In these instances, using models trained in the wrong tissue may lead to poor prediction and misleading TWAS results. GTEx currently has matched genotype and expression data from over 50 tissues, making it a good resource for prediction model training. Further improvements to this reference panel by gathering more samples, especially those from non‐European populations, and by gathering data for tissues and cell types not currently measured in GTEx will make these resources even more valuable to the TWAS community.

In conclusion, we have shown that modeling methods that assume sparsity (as implemented in most transcriptomic imputation software packages) generally achieve the best gene expression prediction accuracy, although the actual prediction accuracy is low for the majority of genes. We have also demonstrated that increasing sample size and careful matching of ancestry and tissue between model training and testing populations improves prediction accuracy. Further increases in sample size and development of population‐specific reference panels across multiple tissues may help to further improve gene expression prediction accuracy and thus improve the power of future TWAS.

## CONFLICT OF INTERESTS

The authors declare that there are no conflict of interests.

## Supporting information

Supporting information

Supporting information

Supporting information

Supporting information

Supporting information

Supporting information

Supporting information

Supporting information

Supporting information

Supporting information

Supporting information

## Data Availability

Data sharing is not applicable to this article as no new data were created or analysed in this study.
