# Supplementary figures and images for: Investigation of prediction accuracy and the impact of sample size, ancestry, and tissue in transcriptome‐wide association studies
Source: Genet Epidemiol. 2020 Mar 19;44(5):425–41. doi: 10.1002/gepi.22290 (PMC8641384; doi:10.1002/gepi.22290)

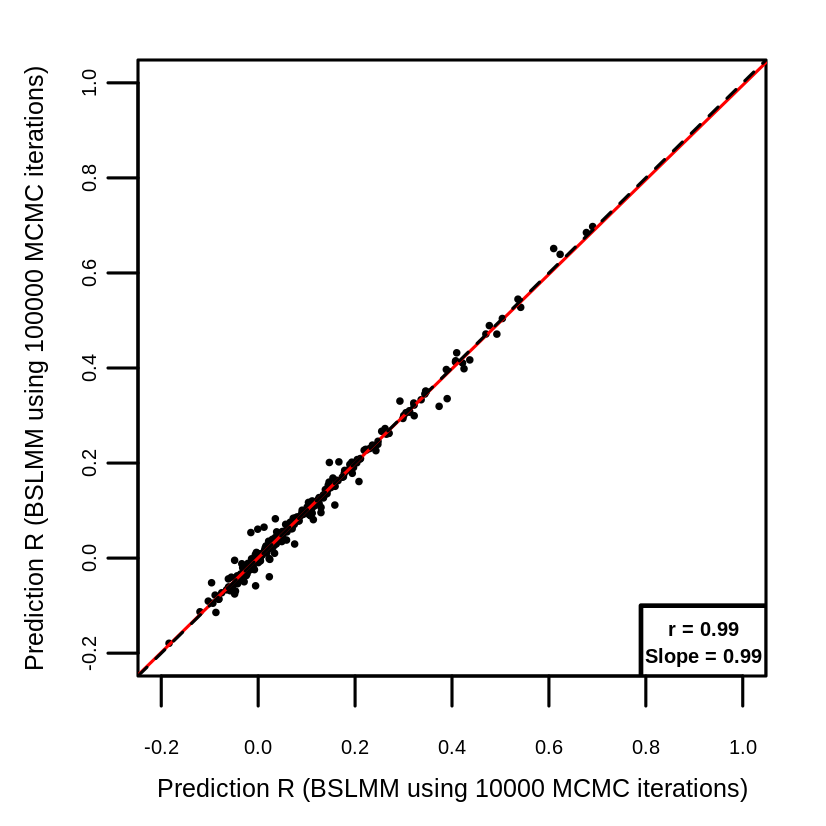

Supplement: Supplementary file 1 — Supporting information [file GEPI-44-425-s009.tif]

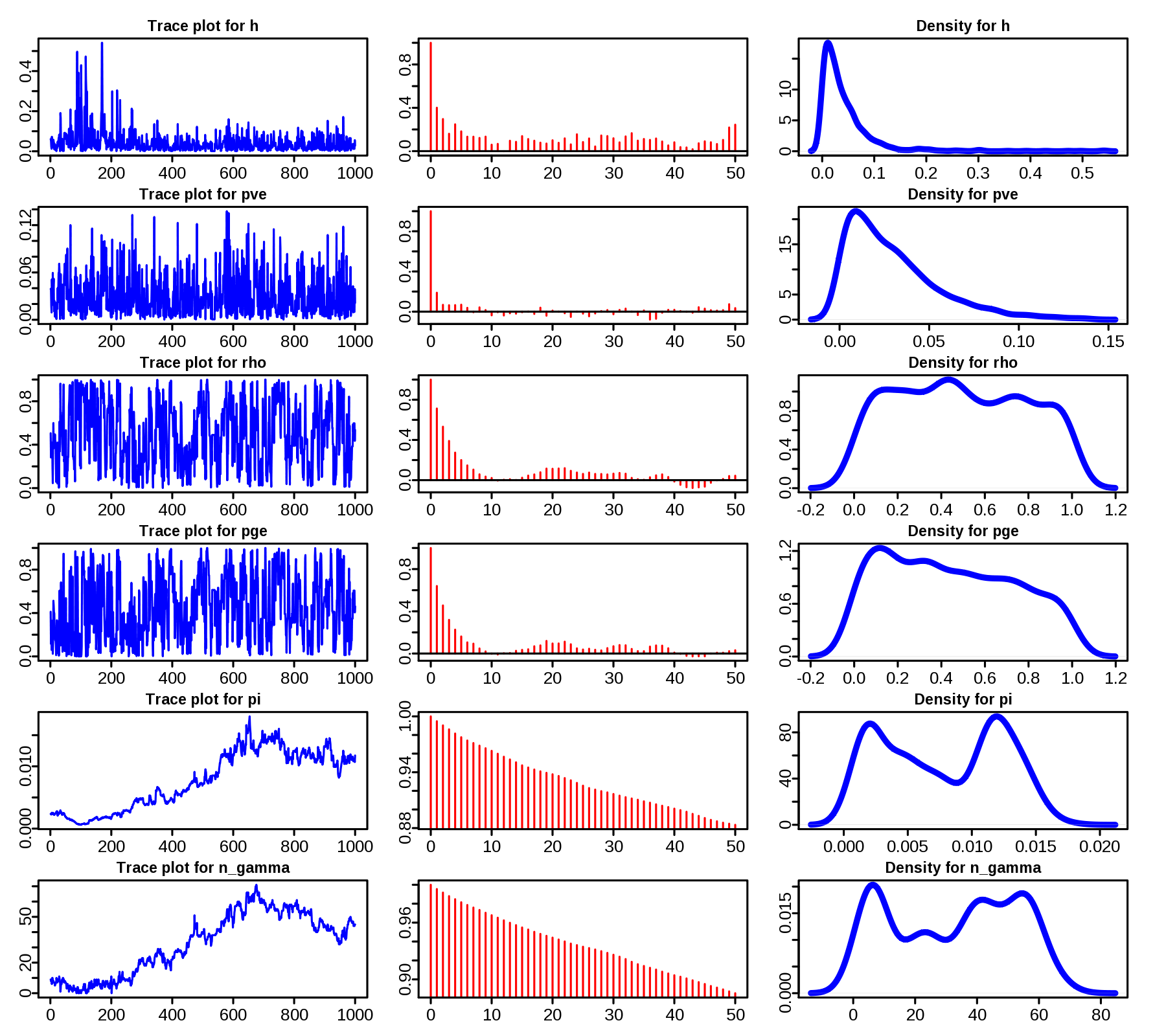

Supplement: Supplementary file 2 — Supporting information [file GEPI-44-425-s008.tif]

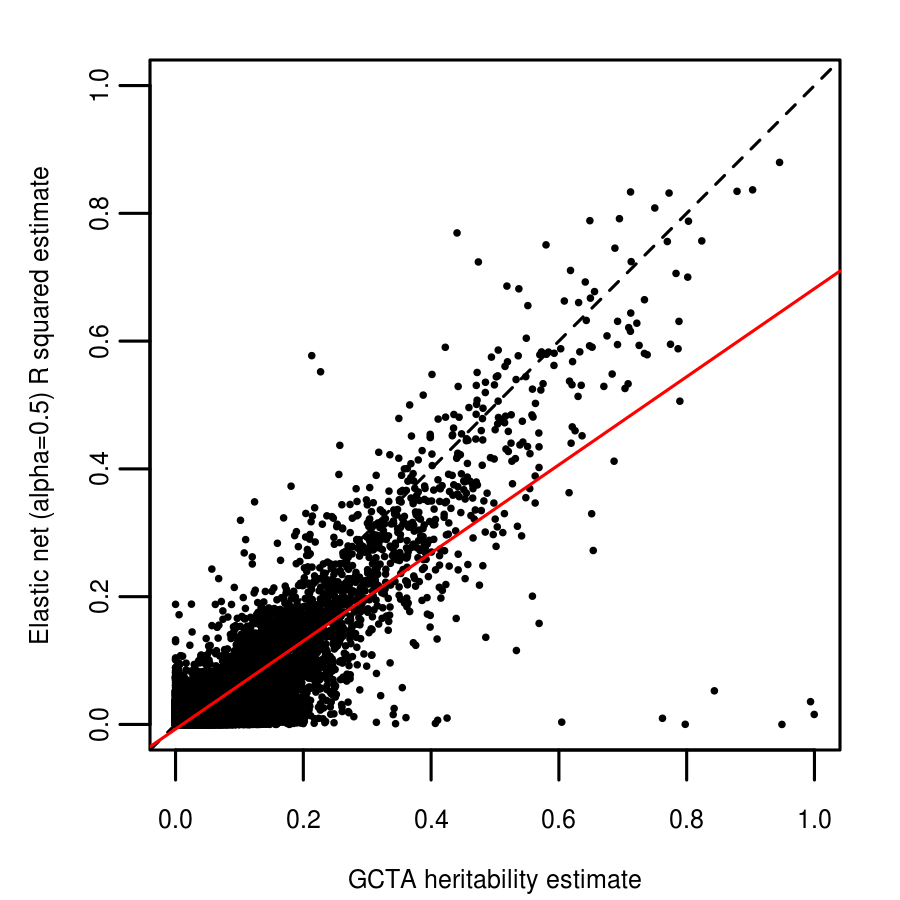

Supplement: Supplementary file 3 — Supporting information [file GEPI-44-425-s005.tif]

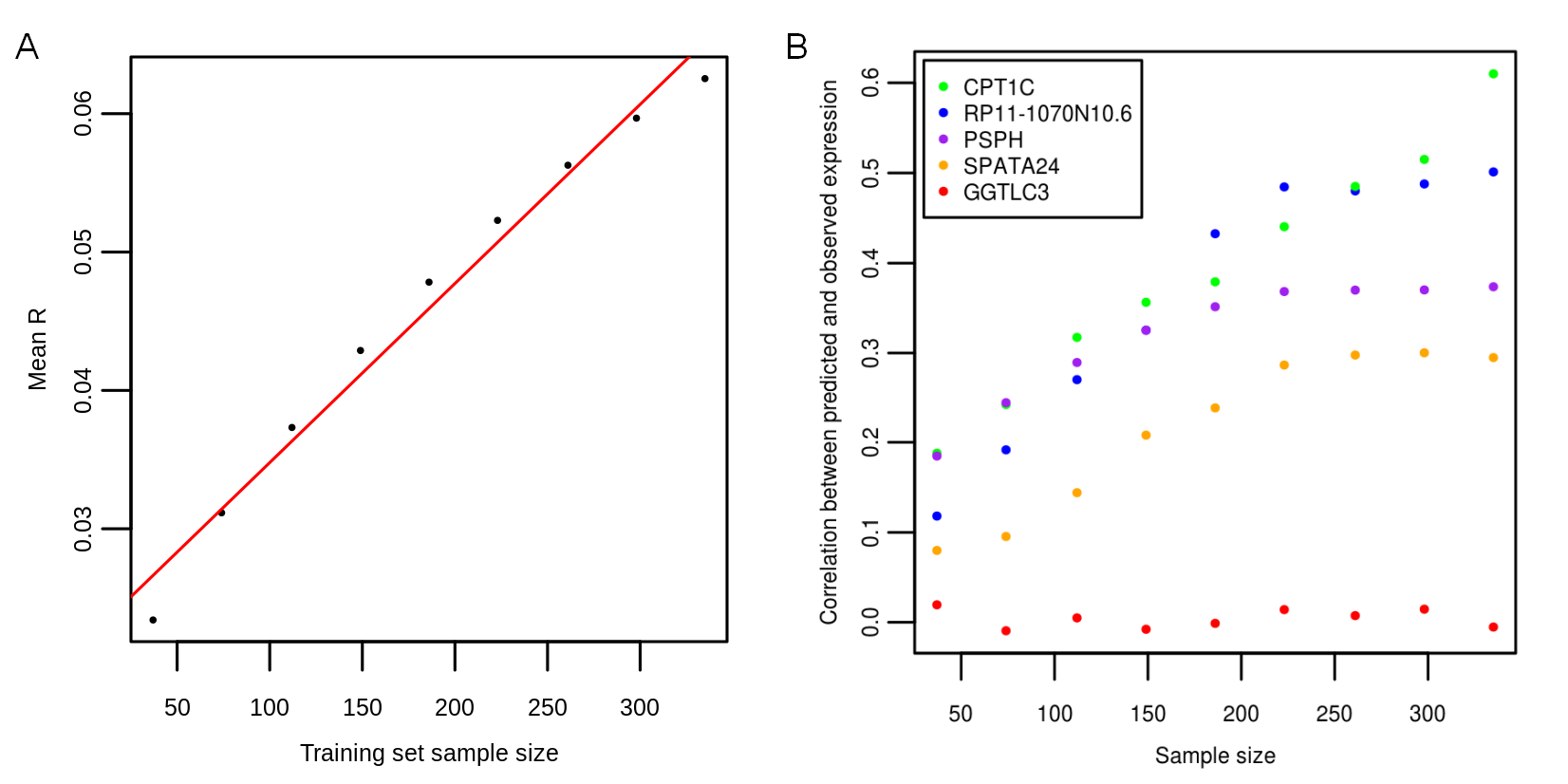

Supplement: Supplementary file 4 — Supporting information [file GEPI-44-425-s010.tif]

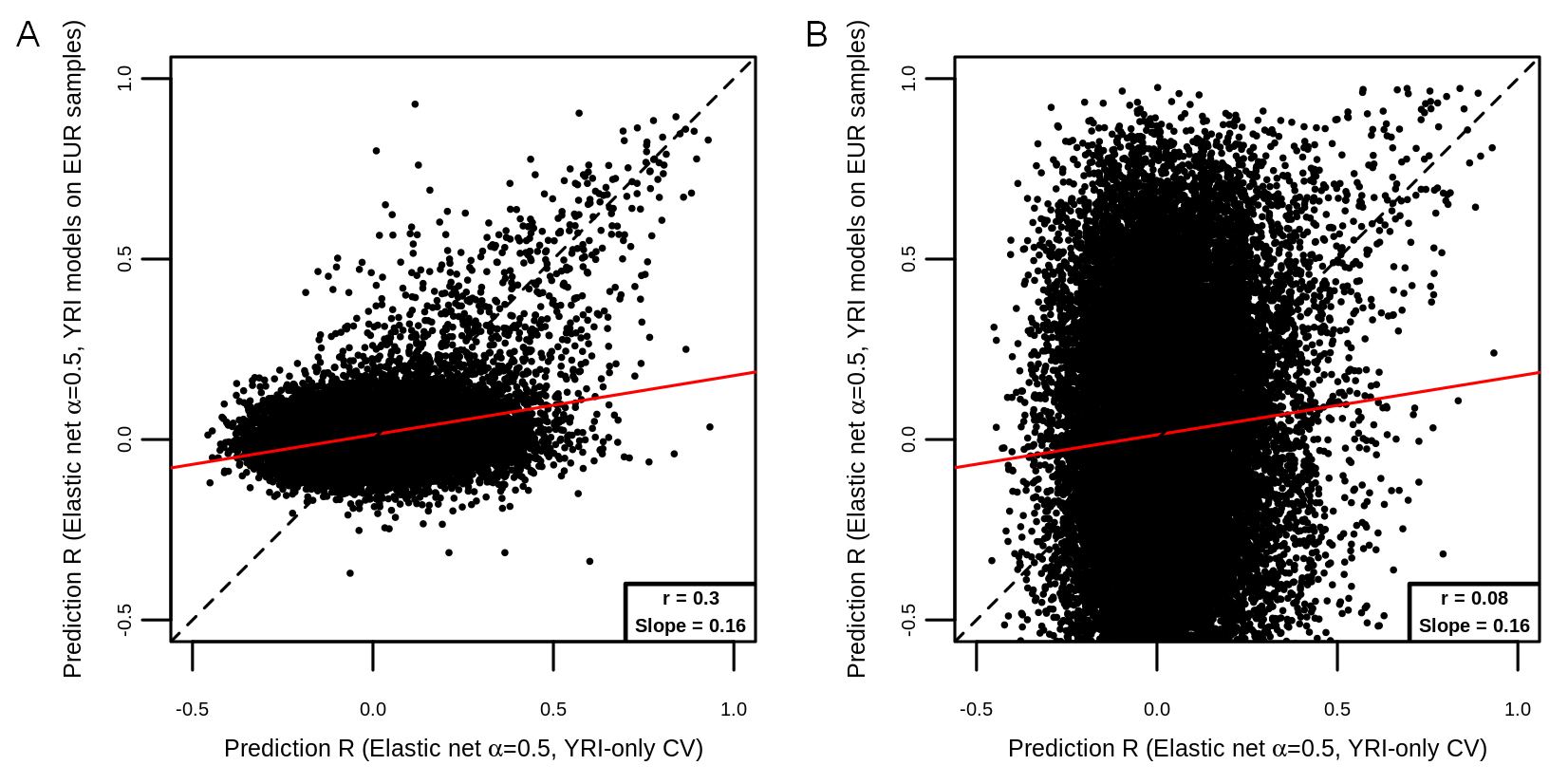

Supplement: Supplementary file 5 — Supporting information [file GEPI-44-425-s001.tif]

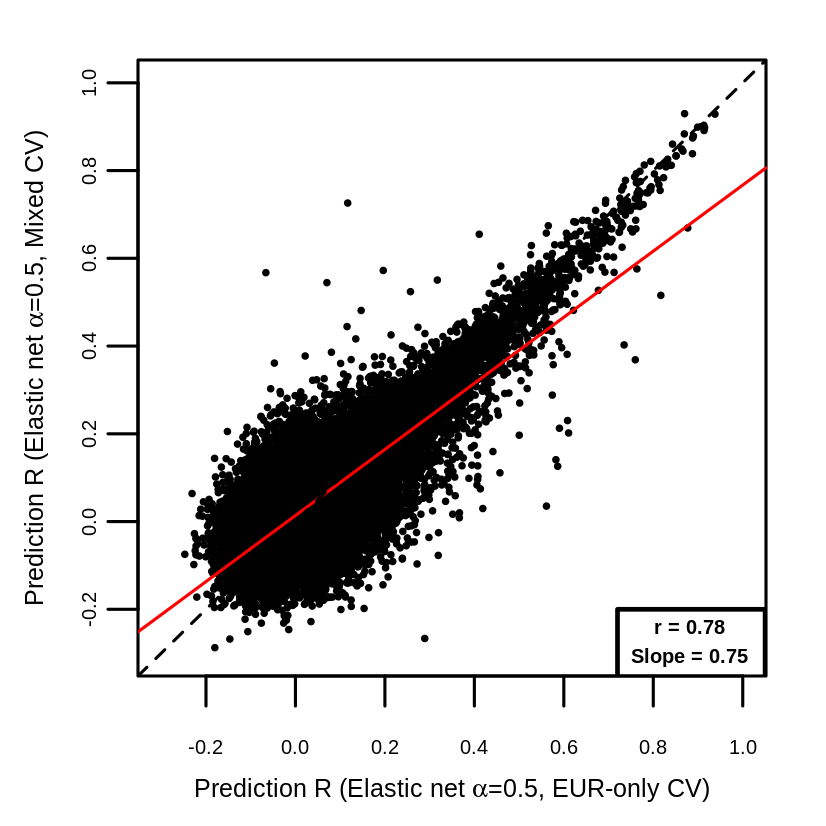

Supplement: Supplementary file 6 — Supporting information [file GEPI-44-425-s002.tif]

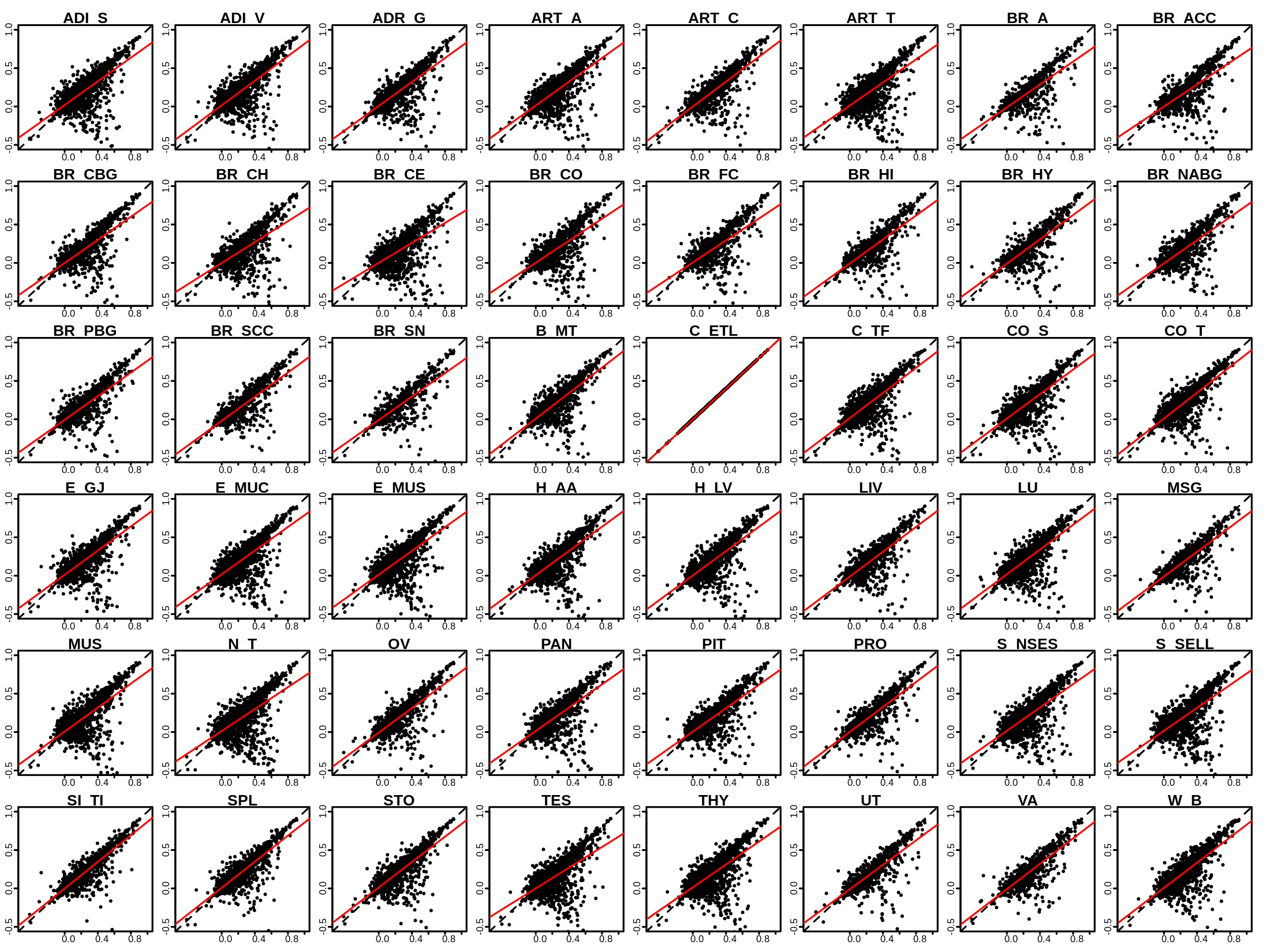

Supplement: Supplementary file 7 — Supporting information [file GEPI-44-425-s003.tif]

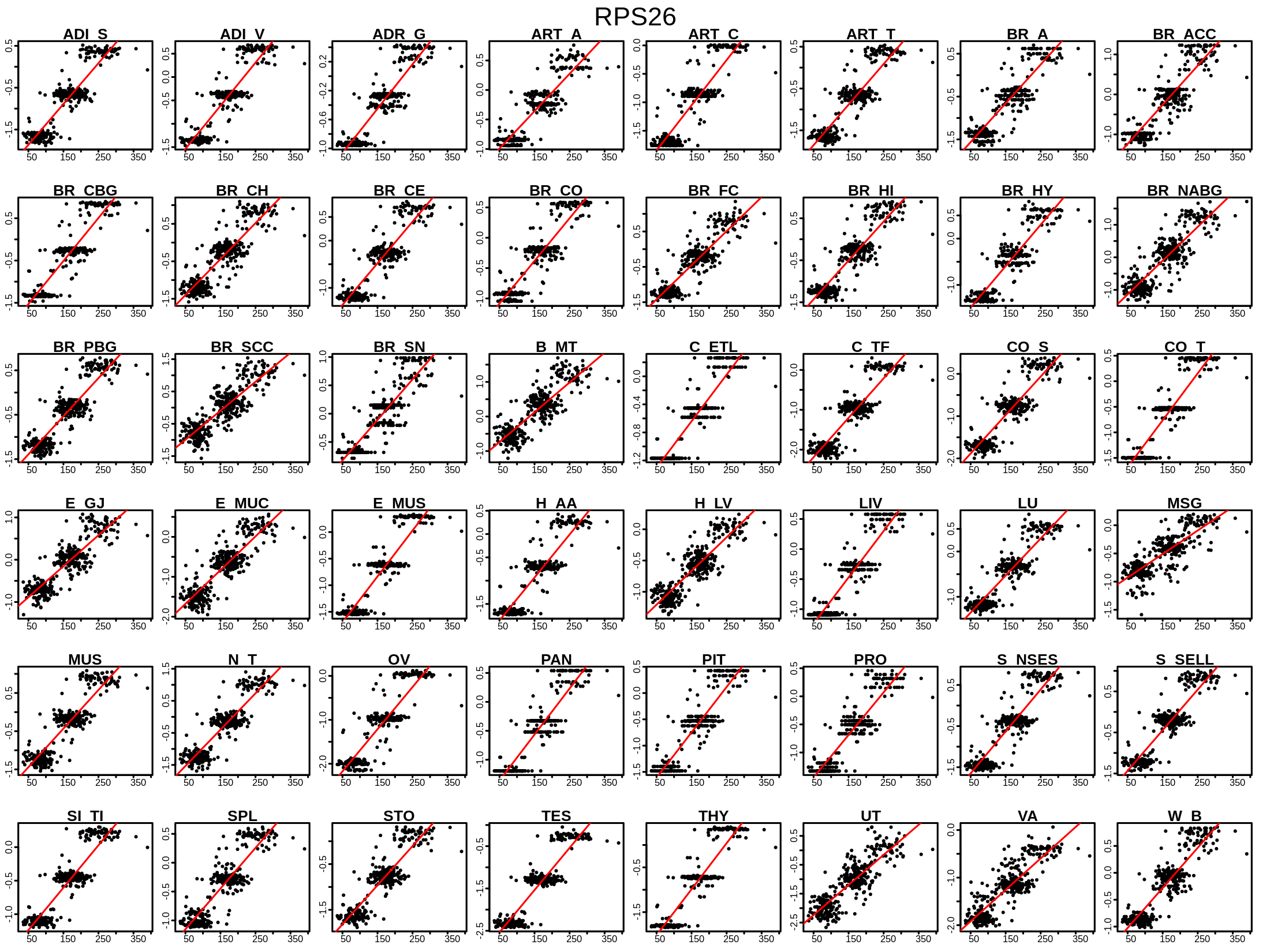

Supplement: Supplementary file 8 — Supporting information [file GEPI-44-425-s004.tif]
