# Supplementary material for: Investigation of prediction accuracy and the impact of sample size, ancestry, and tissue in transcriptome‐wide association studies
Source: Genet Epidemiol. 2020 Mar 19;44(5):425–41. doi: 10.1002/gepi.22290 (PMC8641384; doi:10.1002/gepi.22290)
Supplement: Supplementary file 9 — Supporting information [file GEPI-44-425-s011.docx]

| **Category** | **Gene set** | **Number of genes in set** | **Number of well predicted genes in set** | **p** | **Well predicted genes in set** |
| --- | --- | --- | --- | --- | --- |
| GO_bp | GO_ANTIGEN_PROCESSING_AND_PRESENTATION_OF_ENDOGENOUS_PEPTIDE_ANTIGEN | 14 | 5 | 3.87E-07 | ERAP1:ERAP2:HLA-G:HLA-C:TAP2 |
| GO_bp | GO_INTERFERON_GAMMA_MEDIATED_SIGNALING_PATHWAY | 88 | 9 | 9.30E-07 | TRIM38:HLA-G:HLA-C:HLA-DRB5:HLA-DRB1:HLA-DQA1:HLA-DQB1:HLA-DQA2:HLA-DQB2 |
| GO_bp | GO_ANTIGEN_PROCESSING_AND_PRESENTATION_OF_PEPTIDE_ANTIGEN | 186 | 12 | 2.18E-06 | TAPBPL:ERAP1:ERAP2:HLA-G:HLA-C:HLA-DRB5:HLA-DRB1:HLA-DQA1:HLA-DQB1:HLA-DQA2:HLA-DQB2:TAP2 |
| GO_bp | GO_ANTIGEN_PROCESSING_AND_PRESENTATION | 221 | 13 | 2.32E-06 | TAPBPL:CTSH:ERAP1:ERAP2:HLA-G:HLA-C:HLA-DRB5:HLA-DRB1:HLA-DQA1:HLA-DQB1:HLA-DQA2:HLA-DQB2:TAP2 |
| GO_bp | GO_ANTIGEN_PROCESSING_AND_PRESENTATION_OF_ENDOGENOUS_ANTIGEN | 21 | 5 | 3.68E-06 | ERAP1:ERAP2:HLA-G:HLA-C:TAP2 |
| GO_bp | GO_RRNA_METHYLATION | 27 | 5 | 1.38E-05 | NSUN4:TRMT112:FTSJ3:TRMT61B:TFB1M |
| GO_bp | GO_RESPONSE_TO_INTERFERON_GAMMA | 194 | 11 | 1.95E-05 | RAB20:KIF16B:TRIM38:HLA-G:HLA-C:HLA-DRB5:HLA-DRB1:HLA-DQA1:HLA-DQB1:HLA-DQA2:HLA-DQB2 |
| GWAScatalog | Myositis | 15 | 7 | 1.66E-10 | HLA-C:MICB:HLA-DRB1:HLA-DQA1:HLA-DQB1:HLA-DQA2:HLA-DQB2 |
| GWAScatalog | Pneumonia | 9 | 6 | 1.98E-10 | HLA-C:MICB:HLA-DRB1:HLA-DQA1:HLA-DQB1:HLA-DQB2 |
| GWAScatalog | Lymphoma | 16 | 7 | 2.92E-10 | HLA-DRB5:HLA-DRB1:HLA-DQA1:HLA-DQB1:HLA-DQA2:HLA-DQB2:TAP2 |
| GWAScatalog | Response to hepatitis B vaccine | 22 | 7 | 4.10E-09 | HLA-C:HLA-DQA1:HLA-DQB1:HLA-DQA2:HLA-DQB2:TAP2:HLA-DPB2 |
