# Supplementary material for: Investigation of prediction accuracy and the impact of sample size, ancestry, and tissue in transcriptome‐wide association studies
Source: Genet Epidemiol. 2020 Mar 19;44(5):425–41. doi: 10.1002/gepi.22290 (PMC8641384; doi:10.1002/gepi.22290)
Supplement: Supplementary file 10 — Supporting information [file GEPI-44-425-s006.docx]

| **GTEx tissue** | **Number of genes with predicted tissue expression and measured Geuvadis expression** | **Average correlation between predicted tissue expression and measured Geuvadis expression** |
| --- | --- | --- |
| Adipose_Subcutaneous | 6623 | 0.088605327 |
| Adipose_Visceral_Omentum | 5228 | 0.09848799 |
| Adrenal_Gland | 3748 | 0.097084623 |
| Artery_Aorta | 5415 | 0.089752063 |
| Artery_Coronary | 2802 | 0.104484696 |
| Artery_Tibial | 6758 | 0.080894038 |
| Brain_Amygdala | 1846 | 0.088217435 |
| Brain_Anterior_cingulate_cortex_BA24 | 2586 | 0.088584444 |
| Brain_Caudate_basal_ganglia | 3233 | 0.087732451 |
| Brain_Cerebellar_Hemisphere | 3773 | 0.071577034 |
| Brain_Cerebellum | 4852 | 0.066554543 |
| Brain_Cortex | 3383 | 0.083173707 |
| Brain_Frontal_Cortex_BA9 | 2765 | 0.086790926 |
| Brain_Hippocampus | 2189 | 0.092574144 |
| Brain_Hypothalamus | 2195 | 0.091472362 |
| Brain_Nucleus_accumbens_basal_ganglia | 2778 | 0.088493546 |
| Brain_Putamen_basal_ganglia | 2505 | 0.088153754 |
| Brain_Spinal_cord_cervical_c-1 | 1974 | 0.087679895 |
| Brain_Substantia_nigra | 1581 | 0.090340696 |
| Breast_Mammary_Tissue | 4241 | 0.103683629 |
| Cells_EBV-transformed_lymphocytes | 2737 | 0.187777849 |
| Cells_Transformed_fibroblasts | 6226 | 0.098252952 |
| Colon_Sigmoid | 4211 | 0.099063987 |
| Colon_Transverse | 4406 | 0.109779598 |
| Esophagus_Gastroesophageal_Junction | 4275 | 0.099992389 |
| Esophagus_Mucosa | 6672 | 0.090742269 |
| Esophagus_Muscularis | 6290 | 0.088971649 |
| Heart_Atrial_Appendage | 4811 | 0.09321214 |
| Heart_Left_Ventricle | 4393 | 0.091620973 |
| Liver | 2708 | 0.092580561 |
| Lung | 6186 | 0.095884897 |
| Minor_Salivary_Gland | 1770 | 0.10750102 |
| Muscle_Skeletal | 6263 | 0.078191922 |
| Nerve_Tibial | 7440 | 0.074550379 |
| Ovary | 2379 | 0.096074218 |
| Pancreas | 4328 | 0.094152306 |
| Pituitary | 3655 | 0.089296481 |
| Prostate | 2453 | 0.106962104 |
| Skin_Not_Sun_Exposed_Suprapubic | 6034 | 0.086702432 |
| Skin_Sun_Exposed_Lower_leg | 7142 | 0.081356035 |
| Small_Intestine_Terminal_Ileum | 2443 | 0.120229202 |
| Spleen | 3712 | 0.113608347 |
| Stomach | 3853 | 0.108943194 |
| Testis | 5844 | 0.063427721 |
| Thyroid | 7481 | 0.077160469 |
| Uterus | 1923 | 0.100672119 |
| Vagina | 1857 | 0.108984662 |
| Whole_Blood | 5376 | 0.095856585 |
