# Supplementary material for: Investigation of prediction accuracy and the impact of sample size, ancestry, and tissue in transcriptome‐wide association studies
Source: Genet Epidemiol. 2020 Mar 19;44(5):425–41. doi: 10.1002/gepi.22290 (PMC8641384; doi:10.1002/gepi.22290)
Supplement: Supplementary file 11 — Supporting information [file GEPI-44-425-s007.docx]

**Supplementary Figure 1. Comparison of BSLMM performance at two different MCMC lengths.** R estimates from 10-fold cross-validation on EUR Geuvadis samples using BSLMM with an MCMC length of 10000 (x axis) and an MCMC length of 100000 (y axis) are shown for each gene on chromosome 18. The line of equality (dashed black) and a best fit line (solid red) are also shown.

**Supplementary Figure 2. Convergence of hyperparameters for BSLMM.** This plot shows convergence of BSLMM hyperparameters for the PARP4P3 gene. Each row contains plots for one of the BSLMM hyperparameters (h, pve, rho, pge, pi, n_gamma). The leftmost graphs show trace plots for these hyperparameters, showing the values of the hyperparameter selected in each step of the MCMC. The central plots show autocorrelation. The rightmost plots show the density of hyperparameter values chosen across the MCMC. It is expected that a hyperparameter should show a trace that travels across the parameter space but hovers around a mean, and autocorrelation that quickly approaches zero. This is seen for the first 4 hyperparameters (h, pve, rho and pge), but not the last 2 (pi and n_gamma), indicating a potential failure of convergence.

**Supplementary Figure 3. Comparison of prediction accuracy estimates with heritability.** Each point shows the estimate of the heritability of gene expression attributable to SNPs within 1Mb of the gene obtained using GCTA (x axis) and the prediction accuracy estimate from the 10-fold nested cross-validation using elastic net (α=0.5) (y axis). In this plot we chose to show prediction accuracy as R^2^ rather than R, as the heritability is the upper bound on the estimate of R^2^. Also shown are the line of equality (black dashed) and a line of best fit (red). Most points lie around the line of equality, indicating that prediction accuracy estimates and heritability estimates were broadly similar. The slope of the best fit line is below one, indicating that on average the heritability estimate was slightly greater than the prediction accuracy estimate.

**Supplementary Figure 4. Prediction accuracy estimates at a range of samples sizes.** Prediction models were trained using 10%, 20%, 30% … of EUR samples, and tested on the remaining samples. In plot A, each point shows the mean R across genes (y axis) and the sample size at which models were trained (x axis). The red line indicates a best fit line between x and y. There is a clear increase in average prediction accuracy with increasing sample size. In plot B, each point shows the prediction accuracy estimate (y axis) achieved for the specified gene, and the sample size at which this prediction accuracy estimate was obtained (x axis). For some of the genes, the prediction accuracy estimates do not continue to increase with increasing sample size.

**Supplementary Figure 5. Comparison of prediction accuracy estimates when YRI-trained models are applied to EUR and YRI populations.** On both plots, each point represents a gene, and shown are the R estimate from 10-fold nested cross-validation within YRI samples (x axis), and the R between expression predicted using models trained on YRI samples and applied to EUR samples, and measured EUR expression (y axis). Plot (A) corresponds to the analysis where sample sizes of the model training and testing sets used for the within-YRI analysis was not the same as the sample sizes used in the across-ancestries analysis. Plot (B) corresponds to the analysis where the sample sizes of the model training and testing sets was the same in both the within-YRI and across-ancestries analyses. Also shown are the line of equality (black dashed) and a line of best fit (red solid), with the correlation between x and y and the slope of the best fit line shown in the bottom right corner. Most points lie below the line of equality, and the slope of the best fit line is below 1, indicating that prediction models trained on YRI samples perform better at predicting YRI expression than EUR expression.

**Supplementary Figure 6. Comparison of prediction accuracy estimates when using an EUR-ancestry population and a population of mixed ancestry.** Each point represents a gene, and shown are the R estimate from 10-fold nested cross-validation within EUR samples (x axis), and the R from 10-fold nested cross-validation using the EUR and YRI samples combined into a single group (y axis). Also shown are the line of equality (black dashed) and a line of best fit (red solid), with the correlation between x and y and the slope of the best fit line shown in the bottom right corner. X and y values are highly correlated, but more points lie below the line of equality, and the slope of the best fit line is below 1, indicating that on average, prediction models trained within a population of a single ancestry slightly outperform those trained within a mixed population

**Supplementary Figure 7. Comparison of prediction accuracy achieved by GTEx LCL-trained models and GTEx non-LCL-trained models.** In each plot, the x axis shows the R between measured Geuvadis expression and expression predicted using models trained with GTEx LCL expression data. The y axis in each plot shows the R between measured Geuvadis expression and expression predicted using models trained with GTEx data from a tissue other than LCLs (the tissue is given in the plot sub-heading). Each point represents a gene. In each plot, many points lie near the line of equality, yet a group of points lie far below the line of equality, and the slope of the best fit line is below 1, indicating that GTEx LCL-trained models are able to better predict Geuvadis LCL expression than GTEx non-LCL-trained models on average.

Tissues are: adipose subcutaneous (ADI_S), adipose visceral omentum (ADI_V), adrenal gland (ADR_G), artery aorta (ART_A), artery coronary (ART_C), artery tibial (ART_T), brain – amygdala (BR_A), brain – anterior cingulate cortex (BR_ACC), brain – caudate basal ganglia (BR_CBG), brain – cerebellar hemisphere (BR_CH), brain – cerebellum (BR_CE), brain – cortex (BR_CO), brain – frontal cortex (BR_FC), brain – hippocampus (BR_HI), brain – hypothalamus (BR_HY), brain – nucleus accumbens basal ganglia (BR_NABG), brain – putamen basal ganglia (BR_PBG), brain – spinal cord cervical c-1 (BR_SCC), brain – substantia nigra (BR_SN), breast – mammary tissue (B_MT), cells – LCLs (C_ETL), cells – transformed fibroblasts (C_TF), colon – sigmoid (CO_S), colon – transverse (CO_T), esophagus – gastroesophageal junction (E_GJ), esophagus – mucosa (E_MUC), esophagus – muscularis (E_MUS), heart – atrial appendage (H_AA), heart – left ventricle (H_LV), liver (LIV), lung (LU), minor salivary gland (MSG), muscle – skeletal (MUS), nerve – tibial (N_T), ovary (OV), pancreas (PAN), pituitary (PIT), prostate (PRO), skin – not sun exposed suprapubic (S_NSES), skin – sun exposed lower leg (S_SELL), small intestine – terminal ileum (SI_TI), spleen (SPL), stomach (STO), testis (TES), thyroid (THY), uterus (UT), vagina (VA), whole blood (W_B).

**Supplementary Figure 8. Comparison of predicted RPS26 expression from 48 different GTEx-informed models with measured Geuvadis expression.** Shown are 48 plots, each corresponding to a different GTEx tissue with prediction models available. In each plot, each point shows a Geuvadis EUR sample. On the x axis is the measured RPS26 expression in Geuvadis, and on the y axis is the expression predicted with the prediction model for the labelled tissue. Red lines are lines of best fit between x and y. In each plot, there is a strong positive relationship between predicted and observed expression, indicating that all 48 models perform well at predicting Geuvadis RPS26 expression.

**Supplementary Table 1. Gene set enrichment analysis on 480 well-predicted genes.**

**Supplementary Table 2. Mean R estimates from application of 48 sets of GTEx-trained prediction models to Geuvadis data.**
